# Development and validation for research assessment of Oncotype DX® Breast Recurrence Score, EndoPredict® and Prosigna®

**DOI:** 10.1038/s41523-021-00216-w

**Published:** 2021-02-12

**Authors:** Richard Buus, Zsolt Szijgyarto, Eugene F. Schuster, Hui Xiao, Ben P. Haynes, Ivana Sestak, Jack Cuzick, Laia Paré, Elia Seguí, Nuria Chic, Aleix Prat, Mitch Dowsett, Maggie Chon U. Cheang

**Affiliations:** 1 Breast Cancer Now Toby Robins Research Centre, The Institute of Cancer Research, London, UK; 2 Ralph Lauren Centre for Breast Cancer Research, Royal Marsden Hospital, London, UK; 3 Clinical Trials and Statistics Unit (ICR-CTSU), Division of Clinical Studies, The Institute of Cancer Research, London, UK; 4 Queen Mary University of London, London, UK; 5 Department of Medical Oncology, Hospital Clinic, Barcelona, Spain; 6 Translational Genomics and Targeted Therapies in Solid Tumors, IDIBAPS, Barcelona, Spain

**Keywords:** Breast cancer, Prognostic markers, Tumour biomarkers, Prognostic markers, Translational research

## Abstract

Multi-gene prognostic signatures including the Oncotype® DX Recurrence Score (RS), EndoPredict® (EP) and Prosigna® (Risk Of Recurrence, ROR) are widely used to predict the likelihood of distant recurrence in patients with oestrogen-receptor-positive (ER+), HER2-negative breast cancer. Here, we describe the development and validation of methods to recapitulate RS, EP and ROR scores from NanoString expression data. RNA was available from 107 tumours from postmenopausal women with early-stage, ER+, HER2− breast cancer from the translational Arimidex, Tamoxifen, Alone or in Combination study (TransATAC) where previously these signatures had been assessed with commercial methodology. Gene expression was measured using NanoString nCounter. For RS and EP, conversion factors to adjust for cross-platform variation were estimated using linear regression. For ROR, the steps to perform subgroup-specific normalisation of the gene expression data and calibration factors to calculate the 46-gene ROR score were assessed and verified. Training with bootstrapping (*n* = 59) was followed by validation (*n* = 48) using adjusted, research use only (RUO) NanoString-based algorithms. In the validation set, there was excellent concordance between the RUO scores and their commercial counterparts (*r*_c_(RS) = 0.96, 95% CI 0.93–0.97 with level of agreement (LoA) of −7.69 to 8.12; *r*_c_(EP) = 0.97, 95% CI 0.96–0.98 with LoA of −0.64 to 1.26 and *r*_c_(ROR) = 0.97 (95% CI 0.94–0.98) with LoA of −8.65 to 10.54). There was also a strong agreement in risk stratification: (RS: *κ* = 0.86, *p* < 0.0001; EP: *κ* = 0.87, *p* < 0.0001; ROR: *κ* = 0.92, *p* < 0.001). In conclusion, the calibrated algorithms recapitulate the commercial RS and EP scores on individual biopsies and ROR scores on samples based on subgroup-centreing method using NanoString expression data.

## Introduction

Over 80% of breast cancer patients in the developed western world have oestrogen receptor (ER)-positive disease^1,2^; their treatment normally includes surgery and adjuvant endocrine therapy (ET), and sometimes chemotherapy (CT) which greatly improves outcome^3^. However, a substantial risk remains for relapse.

Multi-parameter gene-expression-based prognostic signatures are often used to estimate the residual risk of recurrence after surgery to guide patient management. Amongst the most widely used prognostic signatures in ER+ breast cancer are the Oncotype DX Recurrence Score (RS)^4^, EndoPredict (EP/EPclin)^5^ and Prosigna® Risk Of Recurrence score (ROR)^6^. Each of these have been endorsed for prognostic use in authoritative guidelines ^7,8^.

RS consists of 16 prognostic genes and five reference genes assessed by RT-PCR. Thirteen of the prognostic genes are grouped into four modules (proliferation, oestrogen, HER2 and invasion), which allow these features to be weighted differently in the signature algorithm with scale between 0 and 100^4^. The HER2 and proliferation modules are thresholded such that quantitative read-outs from those modules are only differentiated in tumours with high expression for the respective modules. The 16 genes were selected from 250 candidates on tumours collected from a mixed cohort of 447 patients collected including the tamoxifen arm of NSABP B-20 trial. The cut-points for individual gene expression were based on the results from NSABP B-20 trial^4^. The final RS algorithm was validated on 668 tamoxifen-treated patients from the NSABP B-14 trial, an ER+, node-negative cohort that contained both HER2-positive and -negative patients. RS does not include clinico-pathological information beyond the molecular score. An RS-pathology-clinical model was developed that improves the prognostic performance of RS^9^, but this seems to be used infrequently in clinical practice. Cut-off points for RS were established to classify patients into low (RS < 18), intermediate (18 ≤ RS ≤ 31) and high risk (RS > 31). The RS has been validated in NSABP B-20^10^, TransATAC^11^ and SWOG 8814^12^. More recently the TAILORx study reported findings for the reduced cut-points of 11 and 26, respectively^13^, showing women with hormone receptor-positive, HER2-negative, axillary node-negative breast cancer, and a high RS of 26 to 100 had better prognosis when treated with ET with adjuvant CT regimens than expected with ET alone; however, there was a lack of CT benefit in patients with RS < 26^13,14^.

The molecular EP score consists of eight prognostic and four reference genes measured by RT-PCR, and ranges between 0 and 15 with a cut-off value of 5 categorising patients into low- and high-risk groups^5^. The clinically applicable EPclin, combines the molecular EP score with tumour size and nodal status where the EPclin value of 3.3 splits patients into low- or high-risk categories^5^. EP and EPclin were trained in an ER+/HER2− population that included patients with both node-negative and -positive disease (*n* = 664). EP and EPclin were validated in ABCSG-6 and -8 trials^5^ and in TransATAC^15^.

Breast cancers can be classified into one of five intrinsic subtypes, namely Luminal A, Luminal B, Basal-like, HER2-enriched and Normal-like by gene expression profiling^16^. The PAM50 algorithm is based on measurement of 50 genes optimally selected to classify the five intrinsic subtypes and eight reference genes^6^. The clinically applied Prosigna® score uses 46 of the 50 intrinsic subtype genes, as measured by the NanoString nCounter, integrated with a proliferation score and tumour size information based on the ROR-PT formula^17,18^ and referred as ROR score here. The ROR score was validated in TransATAC^19^, ABCSG-8^17^ and DBCG cohorts^20^ to predict risk of relapse for postmenopausal women with hormone receptor-positive, node-negative (Stage I or II) or node-positive (Stage II or IIIA) early-stage breast cancer to be treated with adjuvant ET. The final ROR score ranges from 0 to 100 and the risk is calculated differently depending on the nodal status; specifically, node-negative cancers are classified as low (0–40), intermediate (41–60) or high (61–100) risk, 1–3 node-positive cancers are classified as low (0–15), intermediate (16–40) or high (41–100) risk and 4+ node-positive cancers are classed as high risk. The PAM50 genes are measured by the NanoString nCounter which allows the detection of up to 800 RNA species in extracts from formalin-fixed tissues by utilising molecular ‘barcodes’, direct hybridisation, and single molecule imaging without amplification steps that might introduce bias^21^. NanoString is widely used for clinical and biomarker research studies of gene expression in FFPE^22^.

Here, we describe the derivation and validation of RS and EP and ROR scores within ER+/HER2− tumours using gene expression data simultaneously generated by the NanoString nCounter System. We also provide a step-by-step protocol as reference and a guide for computing these scores to allow other researchers to assess the prognostic and predictive value of these scores in research studies.

## Results

### Computation and verification of conversion factors for RS and EP in the training set

The gene-wise conversion factors for RS and EP derived from the TransATAC training cohort (*n* = 59) are listed in Tables 1 and 2, respectively. When applied in the training set (*n* = 59) there were linear relationships between the RS gene expression levels measured by RT-PCR and NanoString for all genes with the exception of *CTSL2* and *GSTM1* due to the lack of expression detected in some samples by the NanoString assay (Supplementary Fig. 1). All the EP genes had linear association between the expression levels measured by RT-PCR and NanoString TransATAC training cohort (*n* = 59) (Supplementary Fig. 2).

**Table 1:** Averaged conversion factors for each gene expression level in the Recurrence Score (RS) signature measured by NanoString.

Gene	*β*_*0,c*_^a^	*β*_*c*_^b^
Proliferation		
* MKI67*	−0.085231	0.911335
* BIRC5*	−1.827968	0.936659
* CCNB1*	−1.116226	0.881016
* MYBL2*	2.025348	0.539252
* AURKA*	3.055030	0.586925
Oestrogen		
* PGR*	0.832910	0.801175
* SCUBE2*	−1.293122	0.979588
* BCL2*	1.700006	0.824674
* ESR1*	0.545113	0.862295
HER2		
* GRB7*	1.049809	0.775301
* ERBB2*	0.207291	0.887745
Invasion		
* MMP11*	−0.316719	0.961213
* CTSL2*	4.093753	0.308125
Other		
* BAG1*	−0.399193	0.872163
* CD68*	1.407529	0.773401
* GSTM1*	6.066959	0.149010

^a^Intercepts.

^b^Slopes.

**Table 2:** Averaged conversion factors for each gene expression level in the EndoPredict (EP) signature measured by NanoString.

Gene	*β*_*0,c*_^a^	*β*_*c*_^b^
*AZGP1*	6.919546	0.986796
*BIRC5*	5.913116	0.940354
*DHCR7*	9.327584	0.939396
*IL6ST*	7.555676	0.942353
*MGP*	3.997335	1.119280
*RBBP8*	7.729633	0.918957
*STC2*	6.418405	0.917461
*UBE2C*	9.732035	0.601117

^a^Intercepts.

^b^Slopes.

### Verification of conversion factors and adjusted expression levels for RS and EP in the validation set

An independent cohort of TransATAC (*n* = 48) was used for the validation of the gene-wise conversion factors. The averaged correction coefficients (Tables 1 and 2) were imputed to adjust the NanoString-derived gene expression levels. Overall, the fit of the adjusted gene expression levels on the commercial gene expression levels for RS and EP was good (Supplementary Figs. 3 and 4). Concordance correlation coefficients (ccc) were >0.85 for all individual RS genes except for *MYBLE2*, *AURKA*, *CTSL2* and *GSTM1* (Supplementary Table 1). Following the adjustment, the expression level of *MYBL2* was overestimated at lower values and underestimated at higher values. Values that were previously undetected by NanoString were shifted from a value of 0 to higher adjusted log2 values, which was noticeable for *CTSL2* and *GSTM1*. For EP, there was a good concordance between the two assays for all EP genes (ccc ≥ 0.84; Supplementary Table 2).

### Evaluation of the RUO RS and EP scores in the validation set

The RS module scores were computed using the algorithms described by Paik et al.^4^. The commercial and adjusted, research use only (RUO) NanoString RS module scores were highly correlated; (ccc) *r*_c_ was 0.91 0.93, 0.98, 0.88, 0.91 and 0.95 for the proliferation, thresholded proliferation, oestrogen, invasion, HER2 and thresholded HER2 modules, respectively (all *p* < 0.0001; Supplementary Fig. 5). There was disparity for four samples in the module thresholdings between the commercial and RUO methods.

**Fig. 1:**
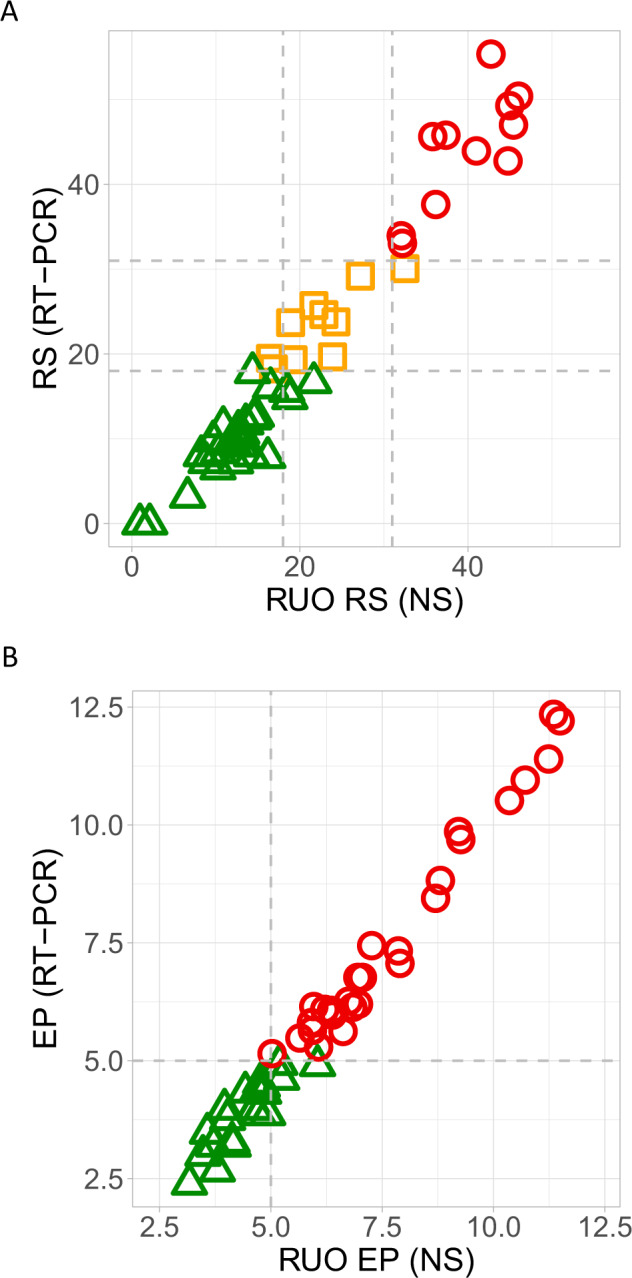
Assessment of relationship between commercial and RUO versions of RS and EP scores. Assessment of correlation (**A**) between commercial RS and RUO RS as well as (**B**) between commercial EP scores and RUO EP scores using the validation set (*n* = 48). Patients are represented in low-risk group with green, in intermediate-risk group with orange and in high-risk group with red as categorised by the commercial tests. RS: Recurrence Score; EP: EndoPredict; NS: NanoString; RUO: research use only.

The ccc for the commercial and RUO RS and EP risk scores were close to one: *r*_c_(RS) = 0.96 (95% CI 0.93–0.97) and *r*_c_(EP) = 0.97 (95% CI 0.96–0.98) (Fig. 1). The high level of agreement between the commercial and RUO RS risk scores was also supported by Bland-Altman plots (Supplementary Fig. 6a, b). For RS, the mean difference between the commercial and RUO RS was Δ(average RS) = 0.21, 95% LoA −7.69 to 8.12. For EP, the mean difference between the commercial and RUO EP scores was Δ(average EP) = 0.31, 95% LoA −0.64 to 1.26.

The validation set samples were classified into risk groups according to the pre-specified cut-points for the RS^4^ and EP^5^ scores. For RS, comparing the commercial and RUO risk groups, of the 48 validation samples, there were 6 (12.5%) cases misclassified by the RUO RS when compared with the commercial RS classification results (Fig. 1a and Table 3). The kappa statistic measuring the agreement between the risk groups defined by the commercial and RUO RS was *κ* = 0.86 (95% CI 0.74–0.97); *p* < 0.0001. Since the report of TAILORx trial results, the cut-offs for RS to identify patients who may benefit to additional CT are 26 for postmenopausal women and 16 for premenopausal, we had repeated our comparisons based on these cut-offs (Supplementary Table 3). The agreements between the commercial and RUO risk groups were substantial with *κ* = 1.00 (95% CI 1.00–1.00) for cut-off at 26 and *κ* = 0.83 (95% CI 0.68–0.99) for cut-off at 16, respectively.

**Table 3:** Classification of 48 validation set patients into risk groups based on the commercial and RUO RS.

Commercial RS (RT-PCR)	RUO RS (NanoString-derived)			
	Low	Intermediate	High	Total
Low	24	3	0	27
Intermediate	2	7	1	10
High	0	0	11	11
Total	26	10	12	48

For EP, there were three patients (3/48, 6.3%) at low risk according to the commercial EP score who were categorised into the high-risk group by the RUO EP (Fig. 1b and Table 4). The kappa statistic measuring the agreement between the commercial and RUO EP risk groups was *κ* = 0.87 (95% CI 0.73–1.00, *p* < 0.0001). The equivalent of the clinically applicable EPclin may be calculated using the EPclin algorithm^5^.

**Table 4:** Classification of 48 validation set patients into risk groups based on the commercial and RUO EP scores.

Commercial EP (RT-PCR)	RUO EP (NanoString-derived) data		Total
	Low	High	
Low	17	3	20
High	0	28	28
Total	17	31	48

### Computation and verification of the RUO ROR

The scaling of the TransATAC dataset to a 229 patient ER+/HER2− cohort assayed previously with the Prosigna® test enabled the robust calculation of subtypes and RUO ROR scores. There was good correlation between the RUO ROR score with the commercial Prosigna® ROR score in the training set (*n* = 59), with ccc *r*_c_(ROR) = 0.95 (95% CI 0.92–0.96) (Supplementary Fig. 7). Fitting a linear regression analysis on these 59 cases, we computed the RUO factors contributing to the formula as set out in Eq. (1):

1 $${\mathrm{RUO}}\;{\mathrm{ROR}} = 12.8536578 + \left( {0.7688329 \times {\mathrm{unadjusted}}\;{\mathrm{ROR}}} \right)$$

**Fig. 2:**
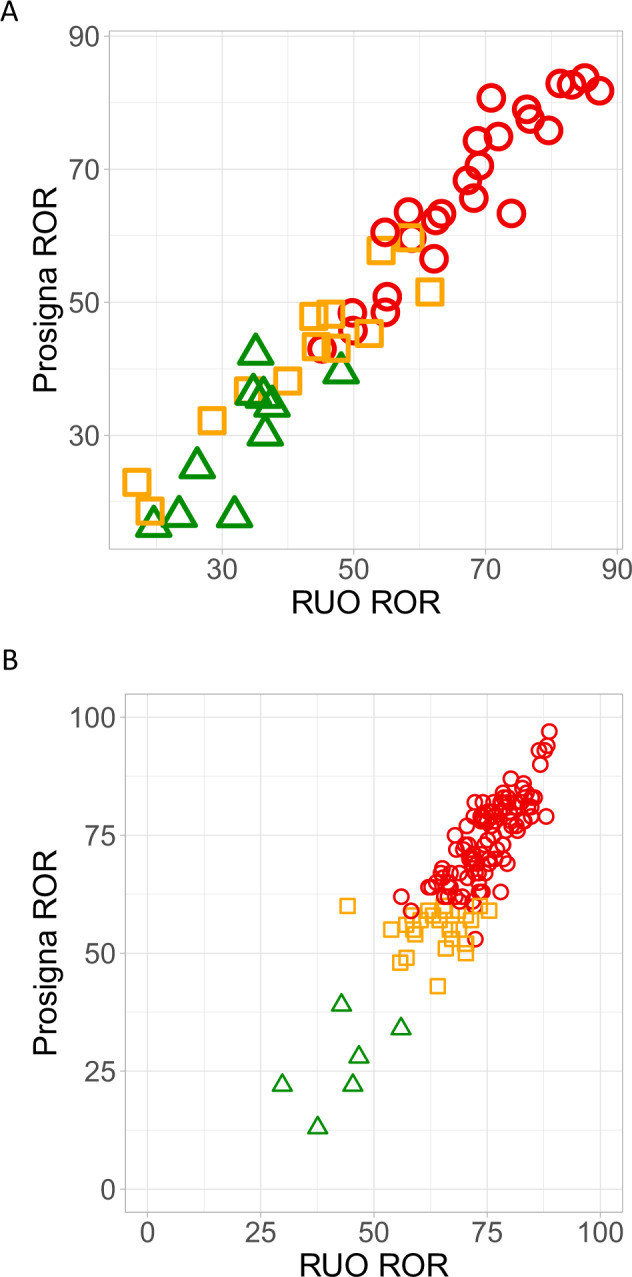
Figure Assessment of relationship between Prosigna® and RUO ROR scores. Comparison of commercial Prosigna® scores and RUO ROR scores (**A**) within the TransATAC validation set (*n* = 48) and (**B**) within the combined Spanish cohort (*n* = 143). Patients are represented in low-risk group with green, in intermediate-risk group with orange and high-risk group with red as stratified by the commercial test. RUO: research use only.

The commercial ROR and RUO ROR scores were highly correlated in the validation set (*n* = 48) with the ccc *r*_c_(ROR) = 0.97 (95% CI 0.94–0.98) (Fig. 2a). The discrepancies between the measurements was visualised using the Bland-Altman plot (Supplementary Fig. 6c) and the bias was low at 0.43 with LoA of −8.65 to 10.54.

Samples were classified into risk groups as set out for the Prosigna®. The kappa statistic measuring the agreement between the commercial and RUO ROR risk groups was *κ* = 0.92 (95% CI 0.84–1.00; *p* < 0.001) in the validation set (Table 5). Nodal status was not available for one patient of the validation set, however, despite this, risk classification was possible due to high commercial and RUO ROR values (>70) both classifying this patient as high risk. There were 3 cases classified differently (6.3%) between these two methods: one node-negative patient was classified as high risk by RUO (RUO ROR = 61.6; luminal B subtype) and intermediate risk by the commercial ROR (ROR = 51.6; luminal B subtype); the second sample was low risk by the RUO (RUO ROR = 35.2; luminal A subtype) and intermediate by the commercial ROR (ROR = 42.3; luminal A subtype); the third sample was intermediate risk by ROR (RUO ROR = 48.1; luminal B subtype) and low risk by the commercial ROR (ROR = 39.4; luminal A subtype).

**Table 5:** Classification of 48 validation set patients into risk groups based on the commercial and RUO ROR scores.

Commercial Prosigna ROR	RUO ROR (NanoString-derived)			Total
	Low	Intermediate	High	
Low	8	1	0	9
Intermediate	1	12	1	14
High	0	0	25	25
Total	9	13	26	48

### Subtype classifications

Of the 59 TransATAC cases within the training set, there were 19 (32.2%) cases defined as Luminal B, 26 (44.1%) as Luminal A, 8 (13.6%) as HER2-enriched and 6 (10.2%) as Normal-like subtypes. Of the 48 TransATAC cases within the validation set, there were 15 (31.3%) cases defined as Luminal B, 14 (29.2%) as Luminal A, 9 (18.8%) as HER2-Enriched, 3 (6.3%) as Basal-like and 7 (14.6%) as Normal-like subtypes. The intrinsic subtype assignments by commercial Prosigna® and RUO ROR were compared for all TransATAC samples (*n* = 107). The Prosigna® test does not use Normal-like subtype for subtype assignment. When a sample is classified as Normal-like subtype, the subtype with the second highest correlation coefficient (usually Luminal A) is used for assignment. Supplementary Table 4 shows the contingency table of subtype calls by the RUO NanoString and Prosigna® methods on the 107 cases, combining the training and validation set, disregarding the Normal-like subtype (i.e. using the second highest correlating subtype if classified Normal-like) with 9 of 58 Prosigna® Luminal A and 14 of 43 Luminal B subtypes classified as other subtypes by the RUO method.

### Estimation on the variability of the RUO ROR algorithms on external gene expression data

A dataset of 146 patients with ER+/HER2− disease from the Hospital Clinic of Barcelona previously tested with Prosigna® and analysed with three different NanoString panels of genes were used for an independent evaluation of the RUO ROR algorithm. In the first cohort of 10 samples, the gene expression data was generated using a custom panel of 110 genes and tumour size information was available for 7 of these samples. The commercial ROR and our RUO ROR scores were highly correlated with ccc r_c_ = 0.85 (95% CI 0.57–0.96) (Supplementary Fig. 8a). In the second cohort of 29 samples, the commercial ROR and our RUO ROR scores were correlated with ccc r_c_ = 0.60 (95% CI 0.38–0.75) (Supplementary Fig. 8b). In the third cohort of 107 samples, the commercial ROR and our RUO ROR scores were correlated with ccc r_c_ = 0.66 (95% CI 0.57–0.74) (Supplementary Fig. 8c). We combined these three cohorts to determine the agreement of risk groups assigned by these two methods. While there was a good agreement between the ROR scores (r_c_ = 0.79 (95% CI 0.74–0.84)) within this cohort of mainly high-risk samples (Fig. 2b), the agreement of categorised risk groups was only fair with a kappa statistics of 0.65 (95% CI –0.45 to 0.86) (Table 6), having 4 cases considered as low risk by commercial ROR but intermediate risk by RUO ROR.

**Table 6:** Classification of 143 external validation cohort patients into risk groups based on the commercial and RUO ROR scores.

Commercial Prosigna ROR	RUO ROR (NanoString-derived)			Total
	Low	Intermediate	High	
Low	2	4	0	6
Intermediate	0	10	17	27
High	0	1	109	110
Total	2	15	126	143

## Discussion

Multi-parameter prognostic signatures are widely used for the prognostication and treatment guidance of ER+/HER2− primary breast cancer patients. We have developed and validated RUO algorithms based on NanoString expression data that closely approximates the commercial prognostic RS, EP and ROR assay scores that are endorsed by international guidelines. In addition, we provided detailed guidelines on how to use the algorithms with the ultimate aim that these can be used for academic clinical research studies, and also how to apply the analytical approach should one wish to compute their own calibration factors internally. A subset of the TransATAC set of samples was selected for this study where the respective prognostic scores had previously been measured by each assays’ commercial developers. Based on the prognostic information available, the cohort was selected to encompass a wide range of recurrence risks.

RS is a purely molecular score without clinical component where we decided to adjust the individual genes. EP, a component of EPclin, is also a molecular score for which the adjustment factors were calculated for individual genes in a similar fashion. For ROR, partly due to the same assay platform being used for the commercial and RUO versions we did not adjust individual genes; instead adjustment to the clinically applicable ROR-PT score was used, an algorithm that also includes a proliferation score and tumour size.

For the RS signature CTSL2 and GSTM1 were not detected in all samples by NanoString. Despite the difficulty in correlating these genes at the lower range of expression, the correlation of individual RS modules and the final score were strong between the commercial RT-PCR-based and RUO NanoString-based data in the validation set, demonstrating the robustness of the RUO RS. For EP there were no gene detection issues and the commercial RT-PCR-based and RUO NanoString-based scores showed a near-perfect agreement in the validation set. For ROR we found good correlation between the final ROR scores obtained by the commercial and RUO methods. There was also good agreement in patient stratification between the commercial and RUO NanoString-based data for all three signatures; this attests that the applied methods adjust well the risk scores obtained by NanoString capturing the information embedded in the commercial risk scores.

There is great interest in computing accurate RS, EP and ROR scores for academic clinical research. Attempts have been made to recapitulate these and other commercial prognostic scores using expression data derived by gene expression microarray^23–25^, RT-PCR^26,27^, RNA-sequencing^25^ and NanoString^28^. However, the surrogate scores are rarely compared to their commercial counterparts derived from the same samples. The main reason is the high cost of the commercial tests which is a substantial impediment in academic studies. The availability of validated signatures at an economically viable cost could facilitate further studies to understand the clinical behaviour of these prognostic gene signatures, predict treatment response and late recurrence, and better understand the impact of different clinical settings on their expression (e.g. the impact of HRT, medical therapies before surgery and phases of the menstrual cycle). Additionally, it would allow the head to head comparative analysis of newly discovered genomic signatures with these three clinically well-established molecular signatures. However, we stress that the presented RUO prognostic scores should not be used for patient management.

Our study has strengths and weaknesses. Strengths include that the samples used in this study are from a well-characterised and documented set of samples. RNA samples have been assayed by the commercial tests’ developers using their commercial proprietary methods used in the clinic. For each patient the same aliquot of RNA was used for the measurements of the commercial assays (RS, EP, ROR) and for the NanoString assay to calculate RUO scores. This eliminated issues related to intratumour heterogeneity and allowed us to calculate more accurate RUO scores. For ROR calculations we used a 229 patient ER+/HER2− scaling cohort which enabled the robust derivation of RUO ROR scores relevant for this TransATAC cohort. The accuracy of conversion factors was confirmed by an independent validation set of 48 samples.

Weaknesses include that the RUO adjustment parameters are optimised for NanoString-based assay, and if working with expression data obtained on different platforms (e.g. RT-PCR, microarray), the normalisation and adjustment factors described here are not applicable. The commercial Prosigna® subtype correlation data and confidence of subtype calls are not available for this TransATAC dataset and it is therefore not possible to accurately determine the robustness of the RUO subtype classification, as samples may have a high confidence correlation with more than one subtype. Meanwhile, our development work had eliminated issues related to intratumour heterogeneity, and the possible variability generated by different RNA quality among a central and a peripheral lab may have been underestimated. In addition, it was a challenge to identify and gain access to external datasets that had been subjected to commercial RS, EP and ROR assays with residual RNA available to assess the accuracy of the conversion factors on data generated by custom gene expression panels in other laboratories. While we were able to assess the variability of ROR scores calculated based on the RUO ROR algorithm using external cohorts from Hospital Clinic of Barcelona, this cohort was biased with much higher risk^29^ than a random population of early ER+/HER2− breast cancer.

A RUO EP and RS can be calculated for individual biopsies; however, for RUO ROR to be calculated for individual samples, improvement is needed for better precision as demonstrated on a testing set of 20 samples (ccc r_c_ = 0.77 (95% CI 0.59–0.88); Supplementary Fig. 9)). Calculation of RUO ROR scores requires generating an ER+/HER2− dataset that roughly matches the cohort of the 229 sample ER+/HER2− tumours used to scale the normalised expression values for each gene. Significant differences between a research dataset and this cohort (e.g. if research dataset is highly enriched for high-risk subtypes) will mean RUO ROR scores will be miscalculated. This weakness might be overcome by the selection of a different cohort for scaling that better matches the dataset of interest or by generating commercial Prosigna® scores for a subset of the dataset that can be used to calculate custom adjustment factors for final RUO scores.

In summary the results presented here show that the RUO NanoString-derived versions of RS, EP and ROR scores closely recapitulate the commercial assessments and may be used to provide high level of discrimination between patients in distinct risk groups as defined by the commercial assays.

## Methods

### Patient samples and study design

The ATAC (anastrozole or tamoxifen alone or in combination) trial evaluated efficacy and safety of anastrozole vs tamoxifen given for 5 years in postmenopausal women with localised primary breast cancer^30^. TransATAC is a collection of patients randomised to the monotherapy arms of the ATAC trial from which 107 ER+/HER2− patients were selected for the current study with the aim to represent low-, intermediate- and high-risk patients where the risk assessments were based on the commercial prognostic tests available in these patients.

Our analytical approach is to compute conversion factors by modelling the gene expression data values measured by NanoString with those assessed by the commercial assays (Supplementary Fig. 10). Stratified by the risk groups, tumour size and nodal status, we randomly split the 107 cases into training set (*n* = 59) for development and validation set (*n* = 48) to determine the correlation coefficient between the RUO and their commercial scores. In order to detect a positive relationship (i.e. correlation >0.4) with 85% power and 0.05 significance level, we would need at least 42 cases. Furthermore, as a secondary analysis, 48 cases would also have approximately 86% power to detect 20% variability of the model at a 0.05 significance level. The Hospital Clinic of Barcelona cohorts are three sets of patient samples with ER+/HER− disease previously tested with Prosigna®. This study was approved by the South-East London Research Ethics Committee and all patients provided written informed consent for their tissue to be used in translational research.

RNA for the TransATAC samples was extracted by Genomic Health Inc. (GHI)^11^. For this study, eligibility required hormone receptor-positive, HER2-negative disease where commercial RS, ROR and EP analyses had been performed by the manufacturer according to the manufacturer protocol using the commercially available test and sufficient amount of residual RNA was available. Measurements of the commercial RS^11^, EP^15^ and ROR^19^ in TransATAC have been described previously^11,15,19^. For each tumour, 150–200 ng RNA was used to measure expression of signature genes constituting the RS, EP and ROR on the nCounter® FLEX Analysis System using a custom gene expression panel of 82 genes. In the current study 72 genes were analysed including 12 reference genes (Supplementary Table 5). For the Hospital Clinic of Barcelona cohorts, a minimum of ∼125 ng of total RNA from formalin-fixed paraffin-embedded tumours (FFPE) were run with one of three different panels: a custom 110 genes panel (*n* = 10), a custom 60 genes panel (*n* = 29) and the NanoString Breast Cancer 360™ panel (*n* = 107).

### Data normalisation procedures

Data normalisation was performed using R version 3.6.3. Raw NanoString output data was normalised using the NanoStringNorm R package^31^.

### Normalisation procedure of training set for RUO RS and EP

For the training set (*n* = 59), the RS and EP signature genes were normalised, respectively, with the following settings:

- CodeCount = ‘geo.mean’
- Background = ‘mean’
- SampleContent = ‘housekeeping.geo.mean’
- round.values = FALSE
- take.log = TRUE
- verbose = TRUE

The commands performed the following: (1) Normalisation of raw data with the geometric mean of the positive controls; (2) Background correction by subtracting the mean of the negative probes; (3) Normalisation with the geometric mean of the housekeeping genes of the respective signature; (4) Setting of <1 values to 1; and (5) Log2 transformation of normalised data.

Additionally, from this set of 59 samples we calculated the average of geometric means of the positive controls before normalisation across the samples (TransATAC-pos) and the average of TransATAC housekeeping geometric means across the samples (TransATAC-hkgm). These parameters were used for the normalisation procedure of the validation set.

### Normalisation procedure of validation set for RUO RS and EP

For the independent validation set of 48 TransATAC samples the RS and EP signature genes were normalised, respectively, with the following parameters:

- CodeCount = ‘geo.mean’
- CodeCount.summary.target = TransATAC-pos*
- Background = ‘mean’
- SampleContent = ‘housekeeping.geo.mean’
- SampleContent.summary.target = TransATAC-hkgm*
- round.values = FALSE
- take.log = TRUE
- verbose = TRUE

These commands performed the following: (1) Normalisation of raw data with the geometric mean of the positive controls setting the target to TransATAC-pos; (2) Background correction by subtracting the mean of the negative probes; (3) Normalisation with the geometric mean of the housekeeping genes of the respective signature setting the target to TransATAC-hkgm; (4) Setting of <1 values to 1; and (5) Log2 transformation of normalised data. *TransATAC-hkgm was 5627.777; the TransATAC-pos for RS was 2461.699 and for EP 8621.201. To utilise the conversion factors described in this study we recommend applying the same normalisation parameters as used for the validation set.

### Normalisation procedure for RUO ROR in the training and validation sets

Raw data was normalised with the following parameters; expression counts plus 1 were log2 transformed and then the geometric mean of the log2 transformed eight housekeeping genes (*ACTB, GUS, MRPL19, PSMC4, PUM1, RPLP0, SF3A1* and *TFRC*) were subtracted from all log2-transformed expression values.

### Estimation of calibration factors — cohort of samples based

Subgroup-specific gene centreing was performed. In brief, the normalised expression data was scaled to a cohort of 229 sample ER+/HER2− tumours previously subjected to the Prosigna® assay (named ERPosHER2Neg) (Supplementary Data 1). Under the assumption that this ER+/HER2− cohort and the TransATAC cohort were similar, gene-wise differences in the median of these two groups represent possible technical and sample bias. To remove such differences, the TransATAC cases were normalised to the ERPosHER2Neg cases. The normalisation factor for each gene was calculated (Supplementary Table 6), and could be applied for other studies of ER-positive tumours. The same calibration factor was applied in the validation cohort (*n* = 48) in this study. The publicly available intrinsic subtype centroids were derived from microarray data, we had scaled our NanoString dataset to a technical calibration factor (Supplementary Table 7) prior to the intrinsic subtype classification^6^.

### Applying the RUO ROR algorithm on Spanish cohorts

The same procedure, as described above, was applied on three set of patients collected externally to assess the agreement of RUO ROR score computed based on our coefficients on different custom NanoString gene panels in other laboratories. The RUO ROR algorithm was applied in each cohort separately as follows. For each cohort, raw data of the 46 ROR genes and 8 housekeeping genes were processed and normalised following the RUO ROR normalisation procedure as described above. Subgroup-specific centreing was performed on the normalised expression data by subtracting the TransATAC-to-ERPosHER2Neg normalisation factor (Supplementary Table 6) for each gene. Intrinsic subtypes were called by applying the PAM50 classifier with the technical calibration factor for NanoString dataset (Supplementary Table 7)^6^. Following the PAM50 subtyping, the ROR score was calculated based on the ROR-PT formula^17,18^. The RUO ROR score was obtained by adjusting the ROR score with the RUO factors.

### Data analysis

#### Development of NanoString RUO RS and EP algorithm

Our analysis pipeline consisted of two steps: (1) estimation of conversion factors in the training set (*n* = 59) and (2) validation of the conversion factors on an independent validation set (*n* = 48) (Supplementary Fig. 10).

#### Estimation of conversion factors in the training set (*n* = 59)

- Computation of conversion factors were repeated 30 times (Iteration, *I* = 30) using a cross-validation approach. The TransATAC training dataset of 59 samples was split into training (sample size, *N* = 39) and test (sample size, *N* = 20) by random sampling (Iteration, *I* = 30). For each iteration, gene-wise conversion factors (intercept and slope) were obtained using linear regression models and were applied to adjust NanoString data using Eq. (2):
  2 $${\mathrm{adjusted}}\,{\mathrm{gene}}_{{\mathrm{NS,}}{\it{zi}}} = \beta _{0,{\it{zi}}} + \left( {\beta _{{\it{zi}}} \times {\mathrm{gene}}_{{\mathrm{NS,}}{\it{zi}}}} \right)$$
  where adjusted gene_NS,*zi*_ is the adjusted *NanoString mRNA* level of gene *(i), β*_*0,zi*_ is the intercept of gene *(i)* and *β*_*zi*_ is the linear coefficient of gene *(i)* in iteration *z* = 1–30.
- The accuracy of conversion factors were evaluated by calculating the % error between the adjusted NanoString gene expression levels and the commercial RT-PCR gene expression level using Eq. (3):
  3 $${\mathrm{Error}}\left( {\mathrm{\% }} \right)_{{\boldsymbol{zi}}} = {\mathrm{median}}\left\{ {\left| {\left( {\frac{{{\mathrm{adjusted}}\;{\mathrm{gene}}_{{\mathrm{NS,}}{\it{zi}}} - {\mathrm{gene}}_{{\mathrm{RTPCR,}}{\it{zi}}}}}{{{\mathrm{gene}}_{{\mathrm{RTPCR,}}{\it{zi}}}}}} \right)} \right| \times 100} \right\}$$
  where adjusted gene_NS,*zi*_ is the adjusted *NanoStringRNA* level of gene *(i)* for the 20 test set samples, gene_RTPCR,*zi*_ is the *RT-PCR mRNA* level of gene *(i)* in iteration *z* = 1–30 for the 20 test set samples.

For each gene the conversion factors (intercept and slope) giving error of <10% were averaged and equate to the final conversion factor using Eq. (4):

4 $$\beta _{0,\,{\it{ci}}} = \frac{{\mathop {\sum }\nolimits_{\it{j}}^{\it{n}} \beta _{{\it{zi}}}}}{{\it{n}}}\,\,{\mathrm{and}}\;{\it{\upbeta }}_{{\it{ci}}} = \frac{{\mathop {\sum }\nolimits_{\it{j}}^{\it{n}} {\it{\upbeta }}_{{\it{zi}}}}}{{\it{n}}}$$

where *β*_*0,ci*_ and *β*_*ci*_ are the average of *β*_*0,zi*_ and *β*_*zi*_ conversion factors giving error of <10% for gene (*i*) in iteration *z* = 1–30, *n* is the number of the coefficients averaged.

#### Validation of the conversion factors on an independent validation set (*n* = 48)

- We applied the final conversion factors (*β*_*0,ci*_ and *β*_*ci*_ of each gene) on the independent validation set (*n* = 48) using Eq. (5):
  5 $${\mathrm{Adjusted}}\,{\mathrm{gene}}\,{\mathrm{expression}}\,{\mathrm{levels}} = {\it{\upbeta }}_{0,\,{\it{ci}}} + \left( {{\it{\upbeta }}_{{\it{ci}}}\,{\it{x}}\,{\mathrm{gene}}_{{\mathrm{NS}}{\it{i}}}} \right)$$
  where *β*_*0,ci*_ and *β*_*ci*_ are the average of conversion factors giving error of accuracy < 10%.
- The RUO RS and EP scores were computed using the adjusted NanoString data and the algorithms reported in the original RS and EP publications^5,10^.

### Development of NanoString RUO ROR algorithm

Following the normalisation and calibration described above, each tumour was assigned to one of the four subtypes based on their similarities as determined by Spearman correlation to the 46 gene-based expression centroids as described by Parker et al.^6,17^. The centroids for the subtypes are provided in Supplementary Table 8 for reference.

Following subtype assignment, the RUO ROR score was calculated using Eq. (6)

6 $$\begin{array}{*{20}{l}}{{\mathrm{ROR}} = 54.7690 {\ast} \left( - 0.0067 {\ast} {\mathrm{Basal}} + 0.4317 {\ast} {\mathrm{HER2E}} - 0.3172 {\ast} {\mathrm{LumA}} + 0.4894 {\ast} {\mathrm{LumB}}\right.}\\ {\left.+ \, 0.1981 {\ast} {\mathrm{Proliferation}}\left( 18 - {\mathrm{gene}} \right) + 0.1133 {\ast} {\mathrm{Tumour}}\;{\mathrm{Size}}\left( {\mathrm{dichotomous}}\;{\mathrm{on}}\;{\mathrm{2cm}} \right) + 0.8826 \right)}\end{array}$$

where the proliferation score is the mean of 18 proliferation genes (*ANLN, CCNE1, CDC20, CDC6, CDCA1, CENPF, CEP55, EXO1, KIF2C, KNTC2, MELK, MKI67, ORC6L, PTTG1, RRM2, TYMS, UCE2C* and *UBE2T*) and tumour size is the pathological tumour size (coded as 0 if ≤2 cm or 1 if >2 cm).

Using the 59 TransATAC cases, we also calculated adjustment factors based on the linear regression Eq. (7):

7 $$\left[\kern-0.15em\left[ {{\mathrm{RUO}}\;{\mathrm{ROR}}} \right]\kern-0.15em\right] = {\it{\upbeta }}0 + ({\it{\upbeta }}1 \times {\mathrm{unadjusted}}\;{\mathrm{ROR}})$$

which was used to adjust the final RUO ROR scores in the validation set.

### Statistical tests

The correlation and agreement between the commercial and RUO prognostic scores were measured by the concordance correlation coefficient and Bland-Altman plot. The agreement between the risk groups defined by the commercial and RUO risk scores was also evaluated with the weighted kappa statistic. STATA and R were used for statistical calculations. Normalisation of NanoString data and image generation were performed with R version 3.6.1.

### Reporting summary

Further information on research design is available in the Nature Research Reporting Summary linked to this article.

## Supplementary information

Supplementary Figures and Tables.

Supplementary Data 1.

Reporting summary.

## Data Availability

The datasets that support the findings of this study are subject to third party restrictions due to contractual agreements, and are therefore not publicly available. The datasets will be made available upon reasonable request. Data access requests are subject to approval, and should be addressed to Dr. Mitch Dowsett, e-mail address: mitchell.dowsett@icr.ac.uk, and Dr. Aleix Prat, e-mail address: alprat@clinic.cat. The data generated and analysed during this study are described in the following metadata record: 10.6084/m9.figshare.13326374.32^32^.

## Code availability

Standard R codes were used. Code requests should be addressed to Dr. Maggie Cheang.
