# Supplementary material for: Development and validation for research assessment of Oncotype DX® Breast Recurrence Score, EndoPredict® and Prosigna®
Source: NPJ Breast Cancer. 2021 Feb 12;7:15. doi: 10.1038/s41523-021-00216-w (PMC7881187; doi:10.1038/s41523-021-00216-w)
Supplement: Supplementary file 1 — Supplementary Figures and Tables. [file 41523_2021_216_MOESM1_ESM.pdf]

Supplementary Figure 1. Scatterplots of RS gene expression levels measured by RT-PCR and NanoString in the training set (n=59). Patients are presented according to risk groups as defined by the commercial RS assay (low risk with green, intermediate risk with orange and high risk with red). NS: NanoString

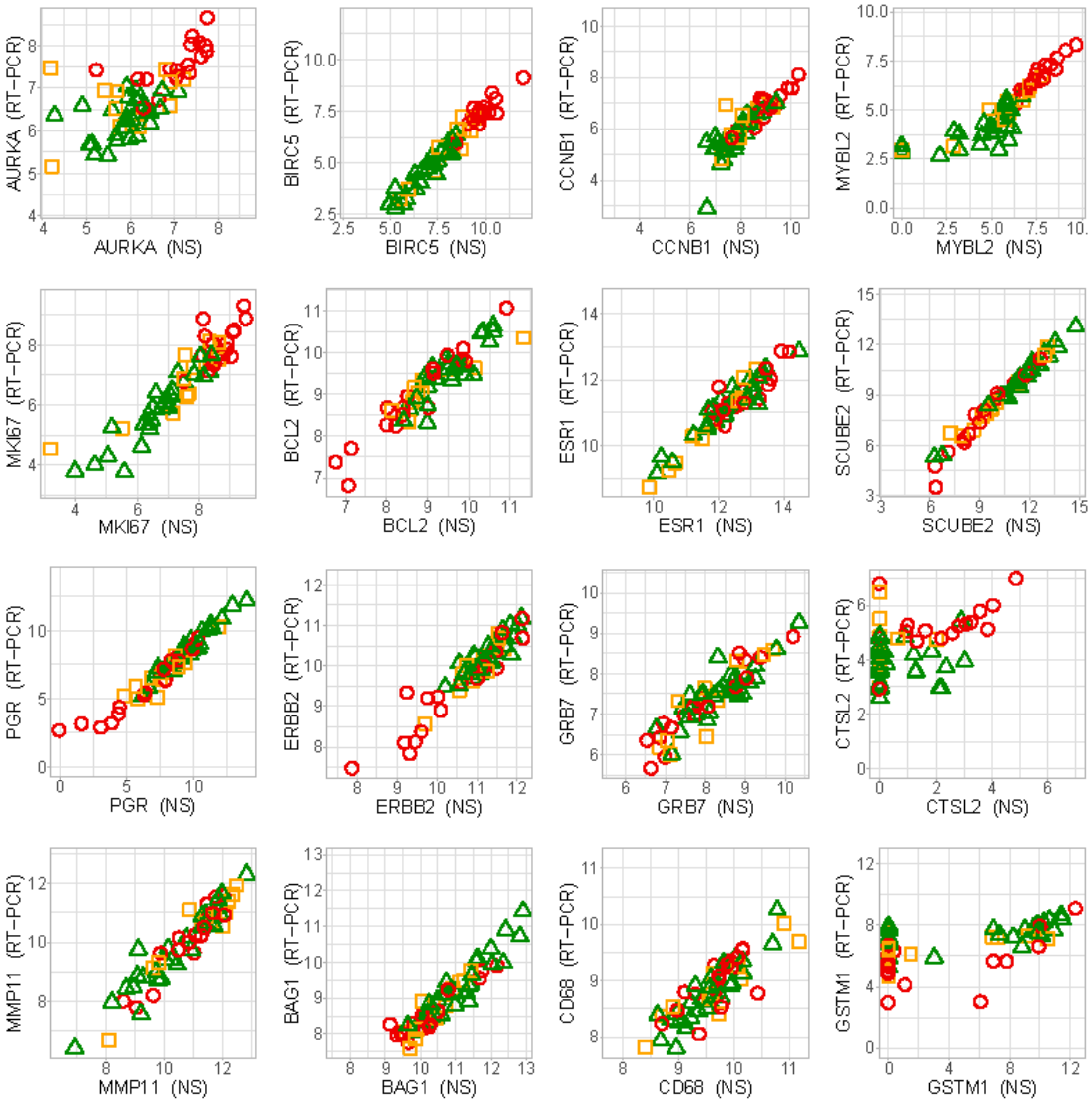

Supplementary Figure 2. Scatterplots of EP gene expression levels measured by RT-PCR and NanoString in the training set (n=59). Patients are presented according to risk groups as defined by the commercial EP assay (low risk with green, high risk with red). NS: NanoString

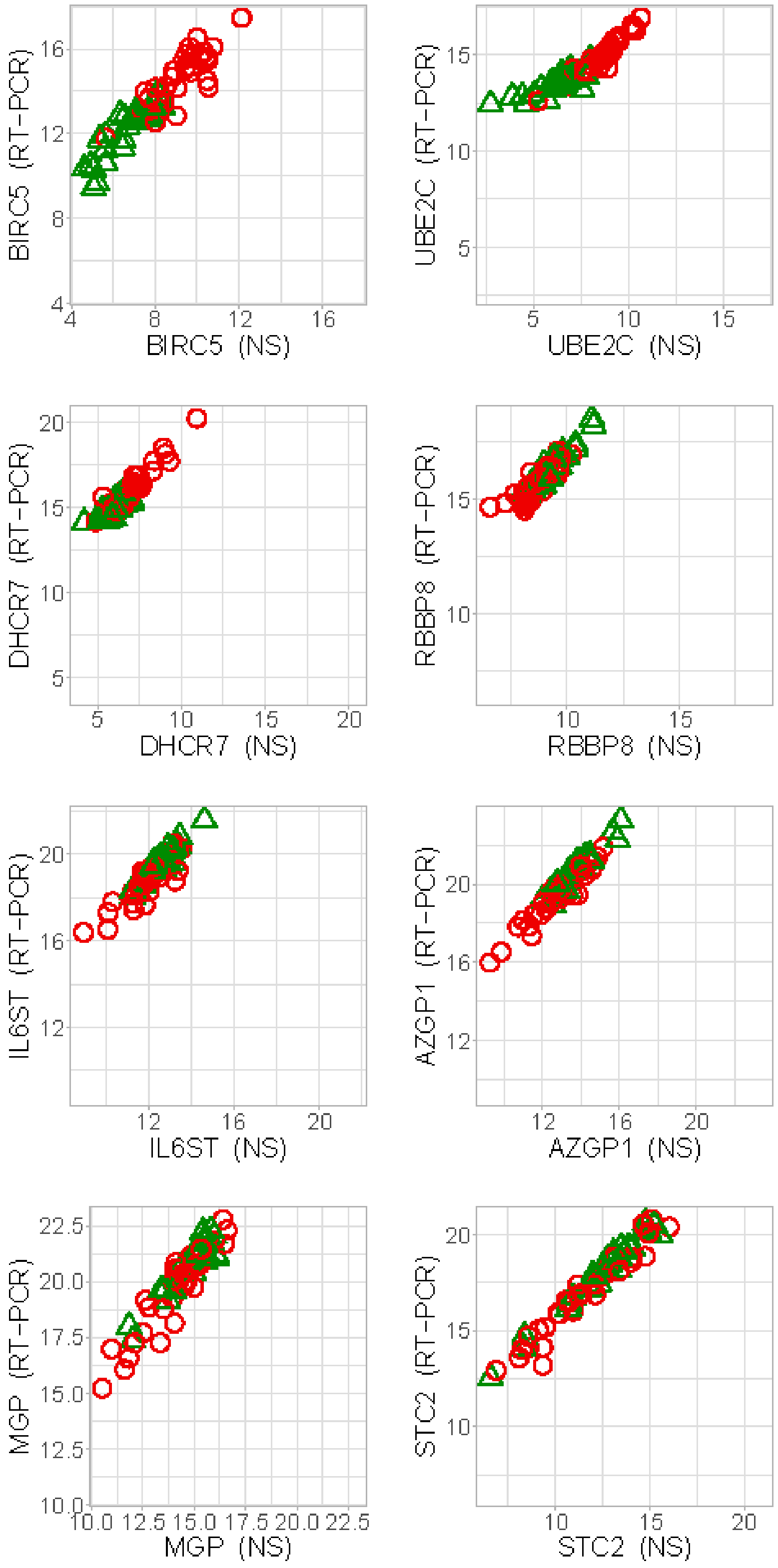

Supplementary Figure 3. Scatterplots of RS gene expression levels measured by RT-PCR and NanoString adjusted by the conversion factors in the validation set (n=48). Patients are presented according to risk groups as defined by the commercial RS assay (low risk with green, intermediate risk with orange and high risk with red). RS: Recurrence Score; adj: adjusted; NS: NanoString

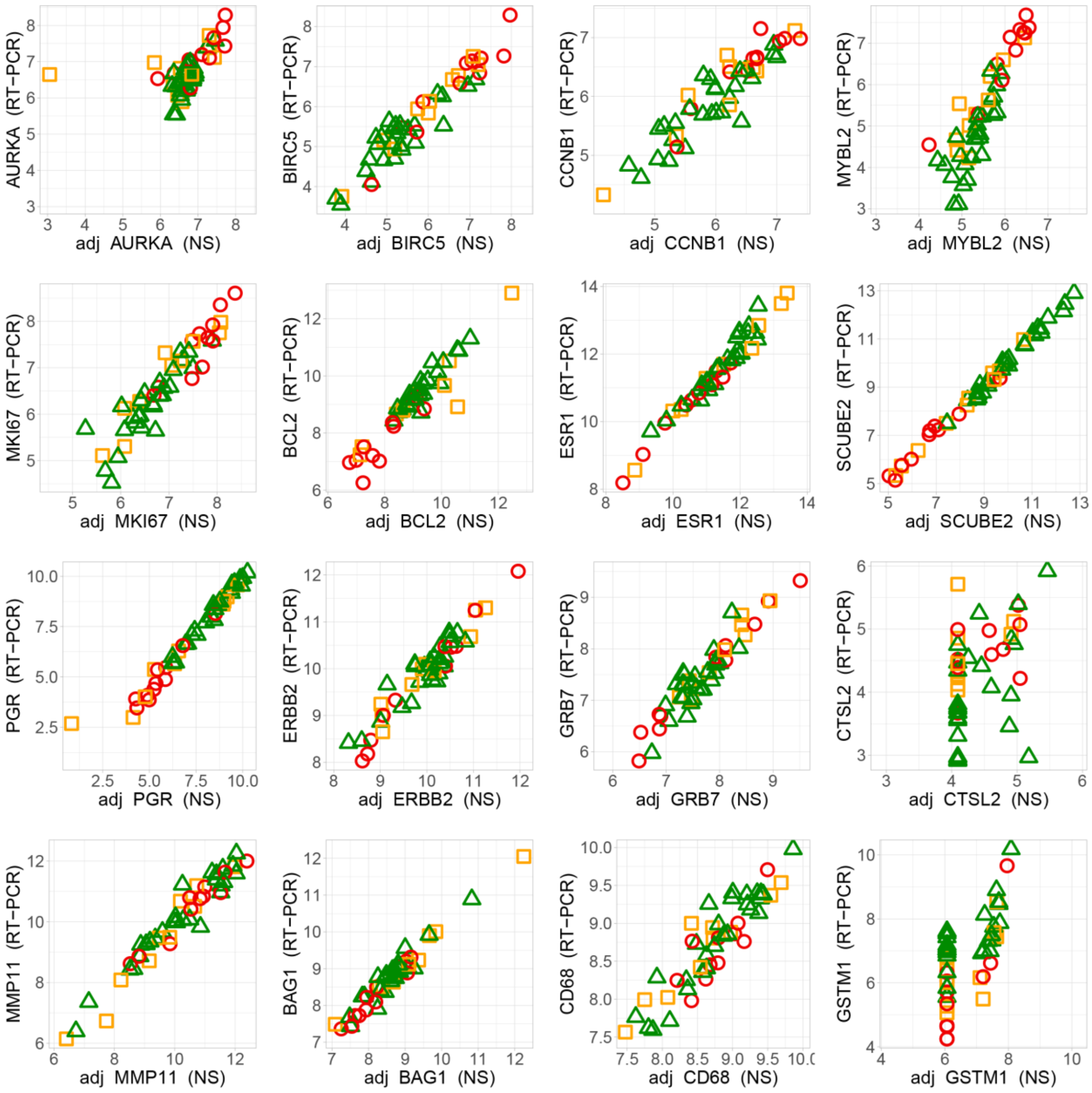

Supplementary Figure 4. Scatterplots of EP gene expression levels measured by RT-PCR and NanoString adjusted by the conversion factors in the validation set (n=48). Patients are presented according to risk groups as defined by the commercial EP assay (low risk with green, high risk with red). adj: adjusted; NS: NanoString

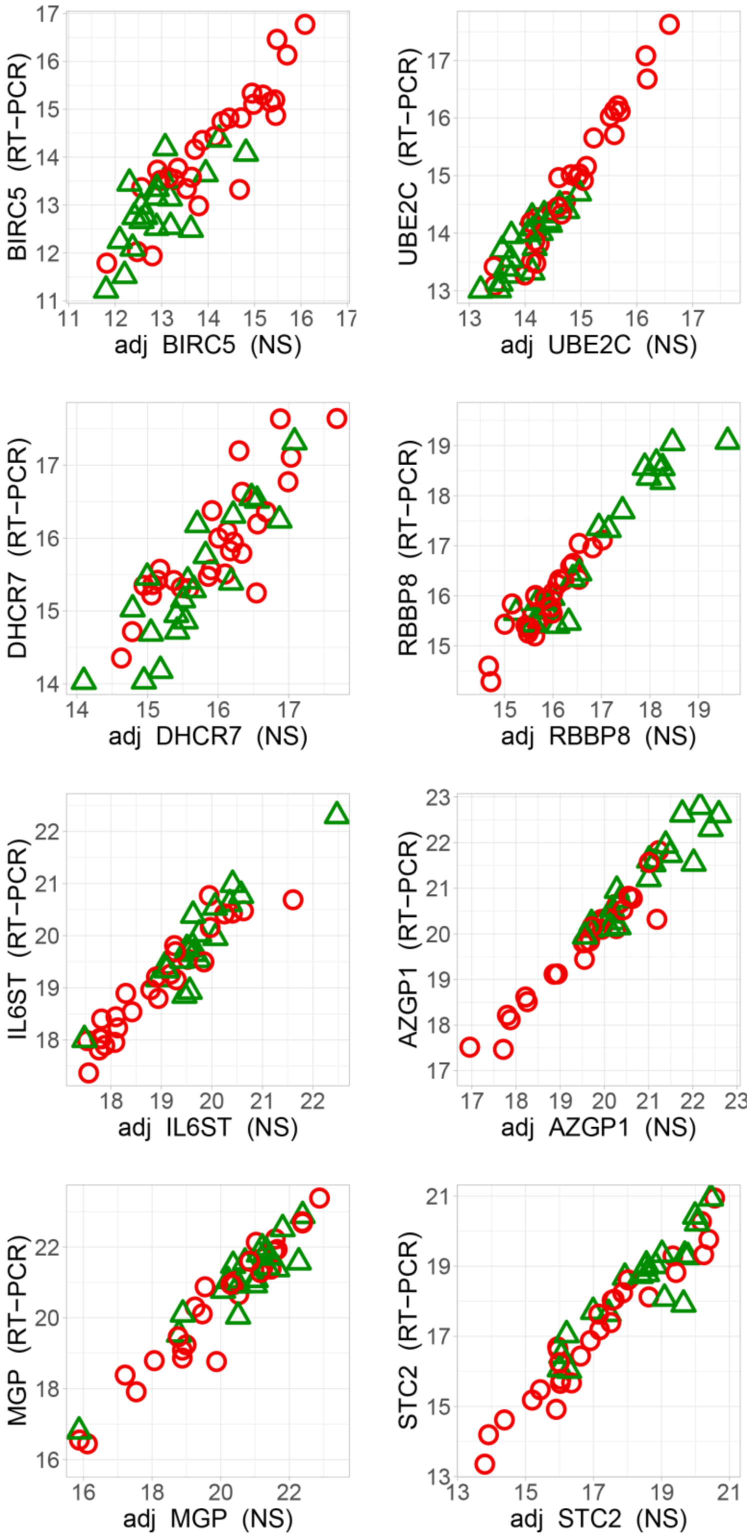

Supplementary Figure 5. Scatterplots of the commercial RS module scores versus NanoString-derived RUO RS module scores in the validation set (n=48). Patients are presented according to risk groups as defined by the commercial RS assay (low risk with green, intermediate risk with orange and high risk with red). Blue lines are the reference lines for the thresholds of the HER2 (threshold = 8) and proliferation (threshold = 6.5) modules.NS: NanoString; mod: module' th: thresholded

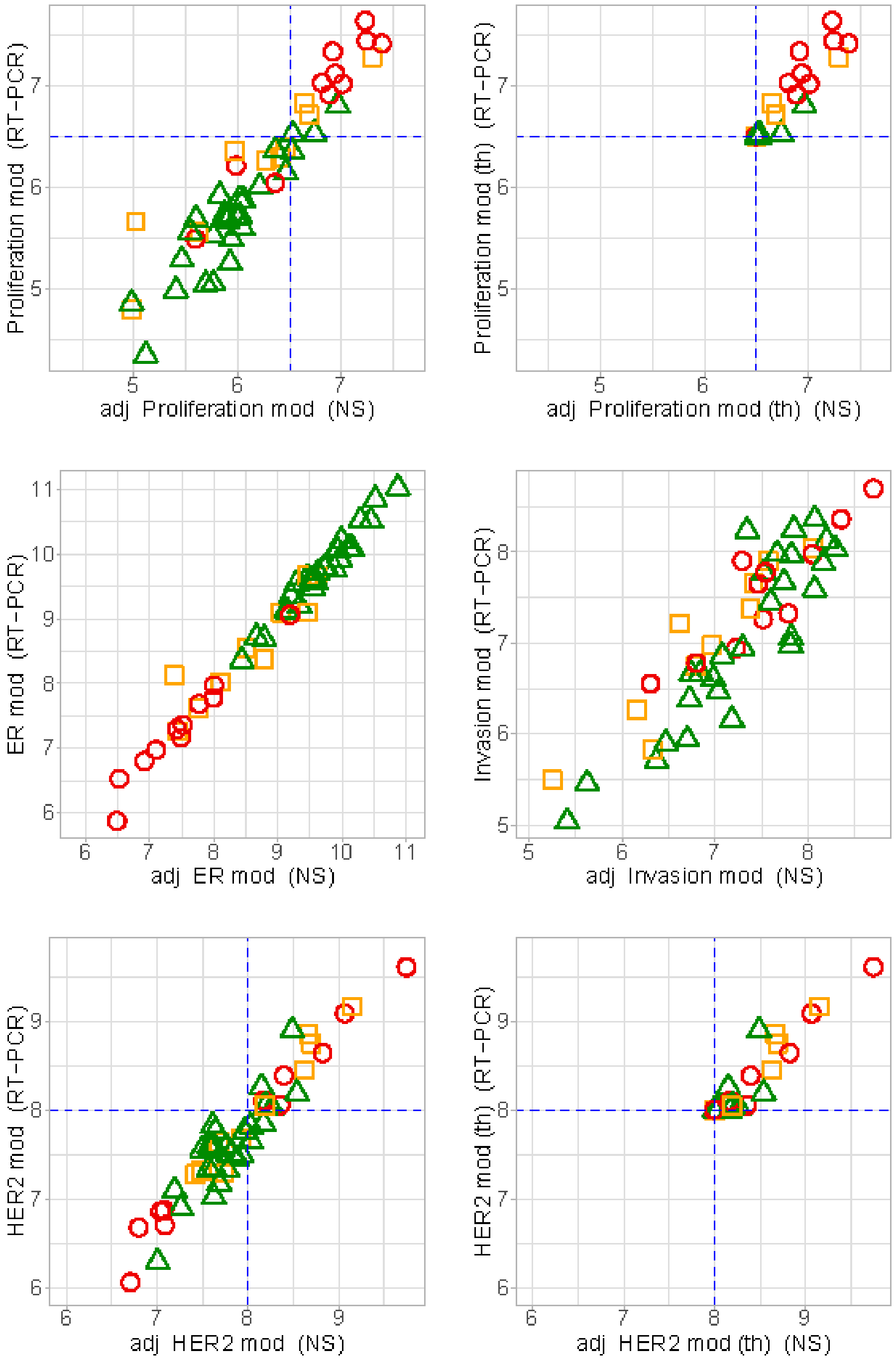

Supplementary Figure 6. Bland-Altman graphical assessment of the agreements between the commercial and RUO (a) RS, (b) EP and (c) ROR scores in the validation set (n = 48).

(a)

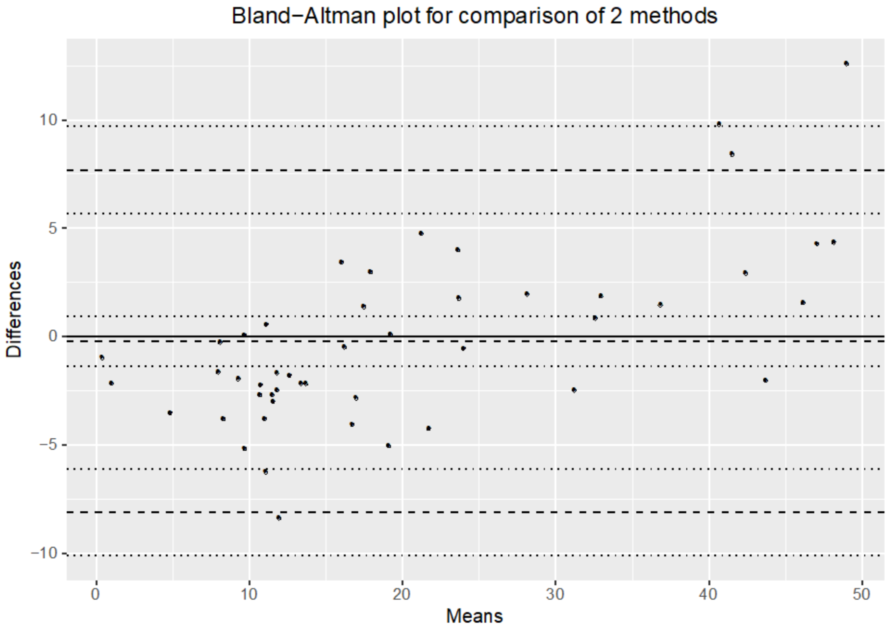

(b)

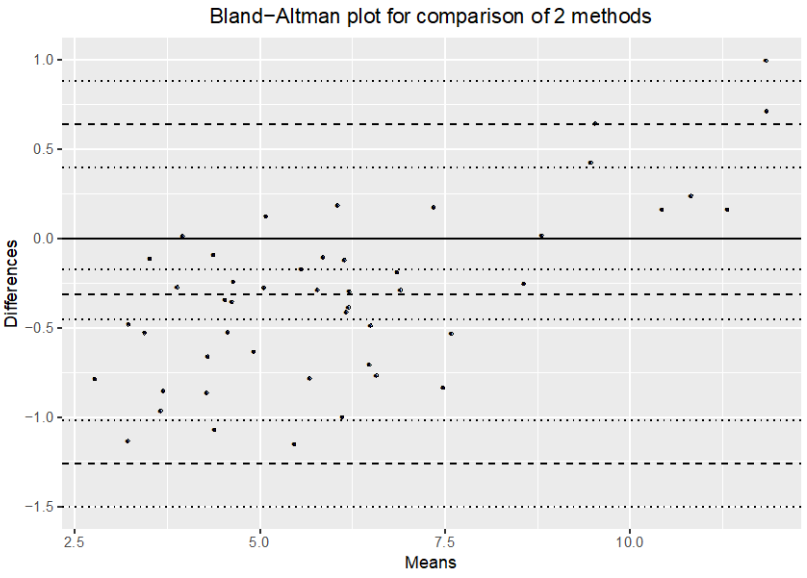

(c)

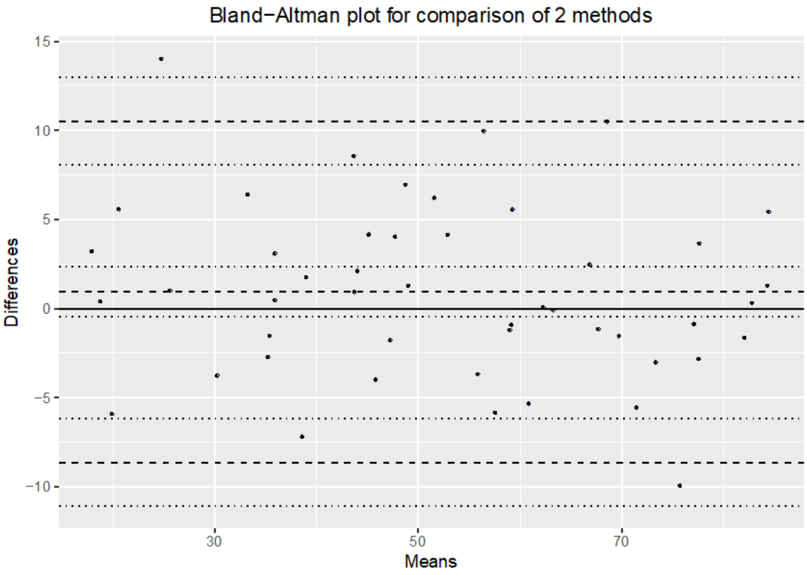
Bland−Altman plot for comparison of 2 methods
15
10
5
0
−5
−10
Differences
30
50
70
Means

Supplementary Figure 7. Assessment of correlation between commercial and RUO ROR scores in the training set (n=59). Patients are presented in low-risk group with green, in intermediate-risk group with orange and high-risk group with red as categorised by the commercial test. RUO: research use only

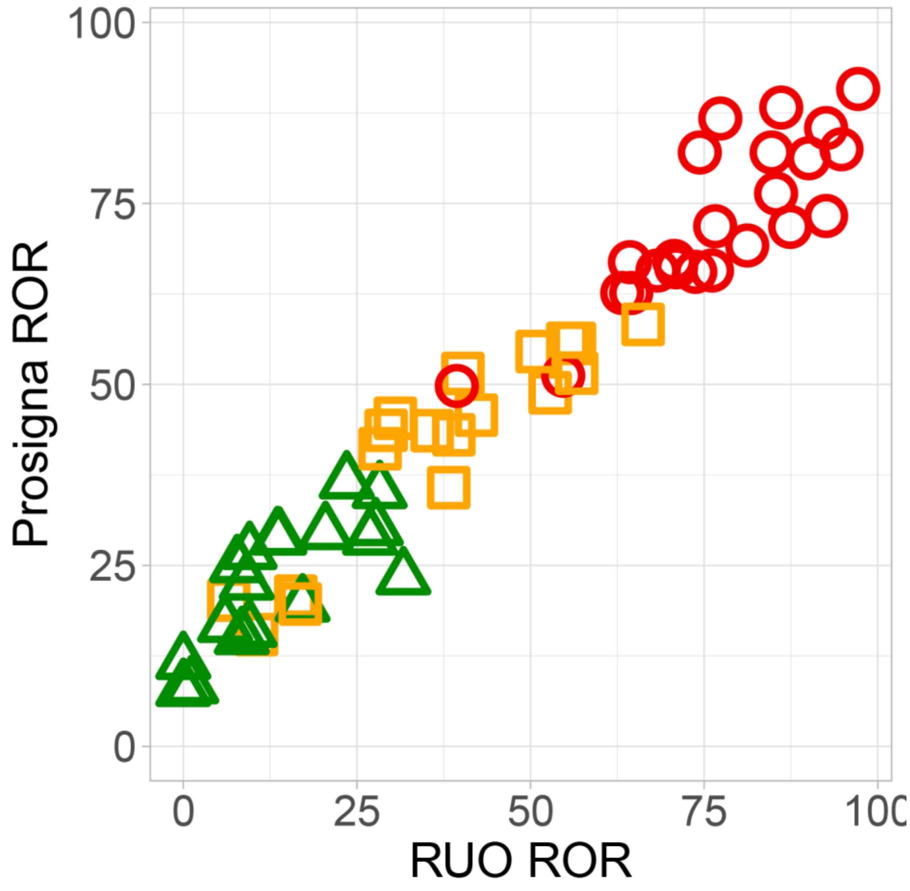

Supplementary Figure 8. Assessment of correlation between commercial and RUO ROR (a) in 7 (b) in 29 and (c) in 107 samples of the Spanish cohort. Patients are presented in low-risk group with green, in intermediate-risk group with orange and high-risk group with red as categorised by the commercial test. RUO: research use only

(a)

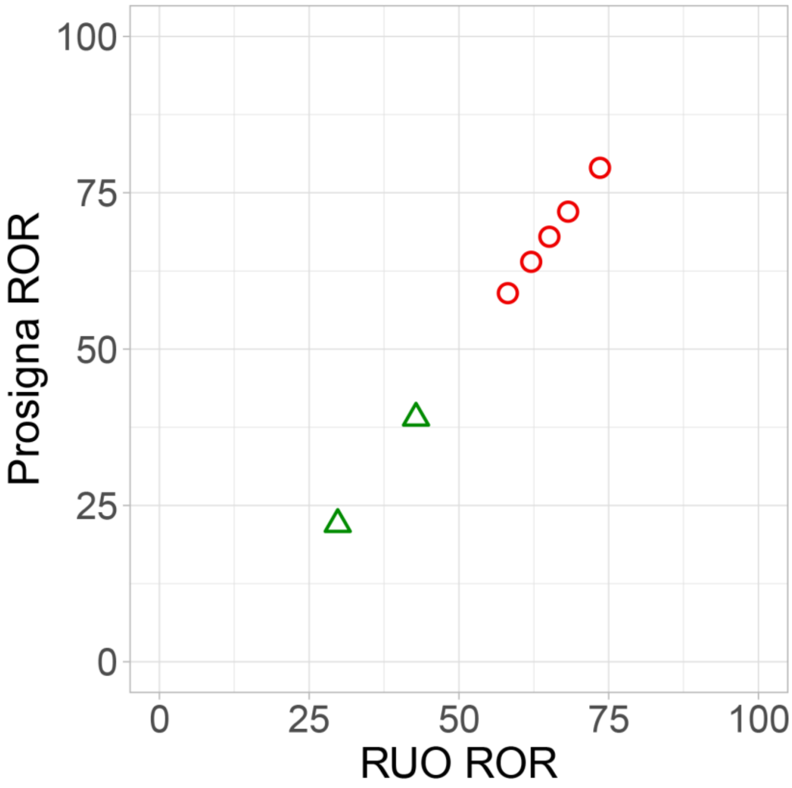

(b)

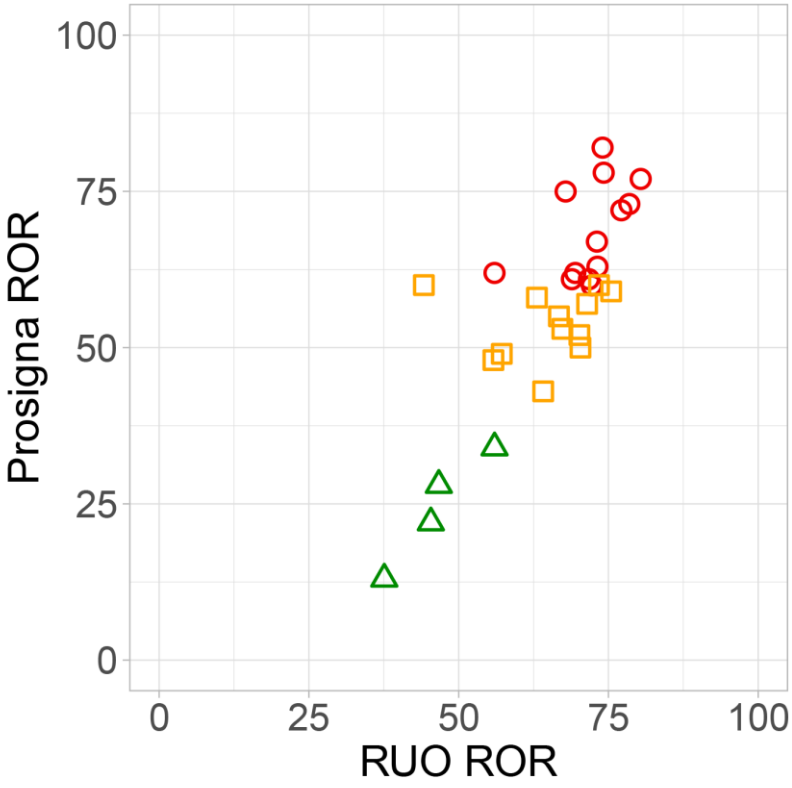
100
75
50
25
0
Prosigna ROR
0
25
50
75
100
RUO ROR

(c)

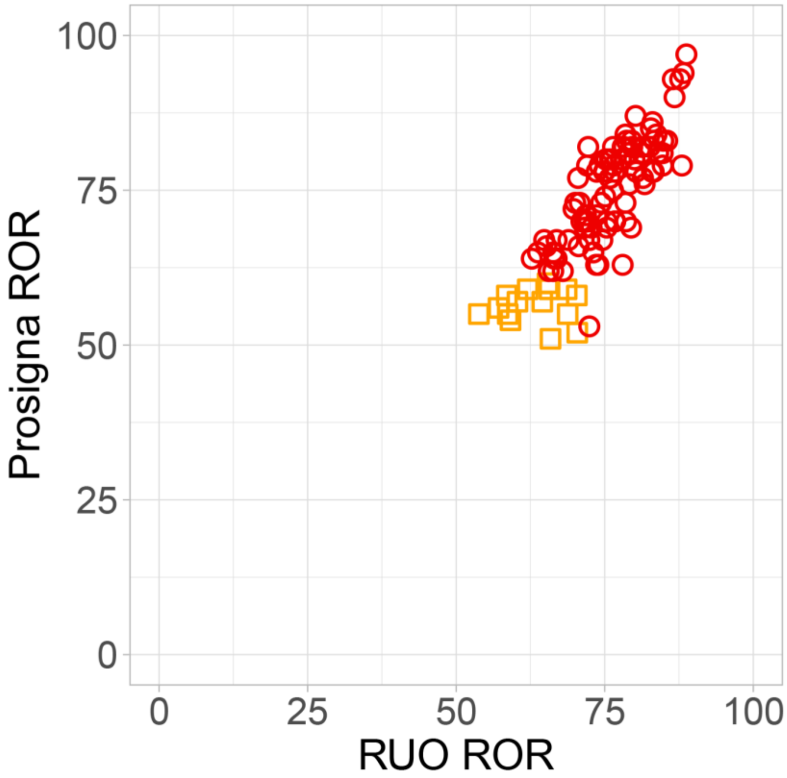
100
75
50
25
0
Prosigna ROR
0
25
50
75
100
RUO ROR

Supplementary Figure 9. Assessment of correlation between commercial and RUO ROR scores as single sample predictor (n=20). Patients are presented in low-risk group with green, in intermediate-risk group with orange and high-risk group with red as categorised by the commercial test. RUO: research use only

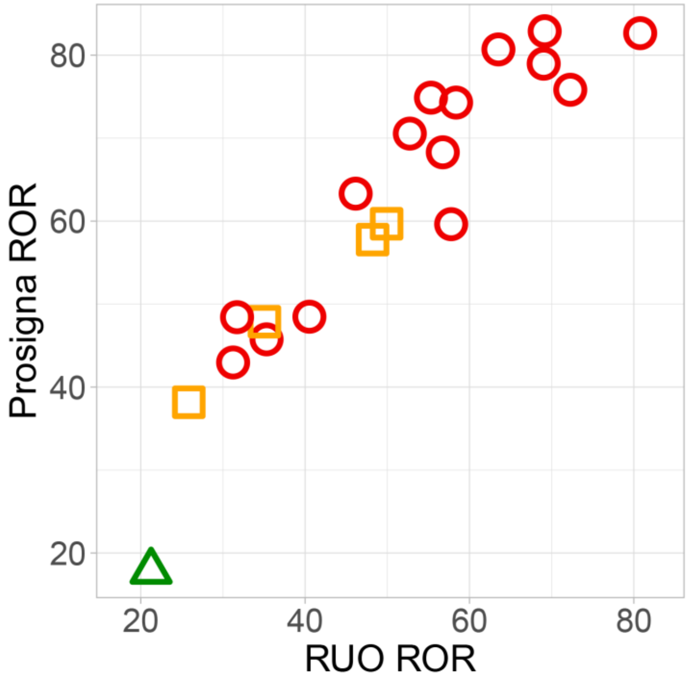

Supplementary Figure 10. Study workflow: computation and validation of conversion methodologies

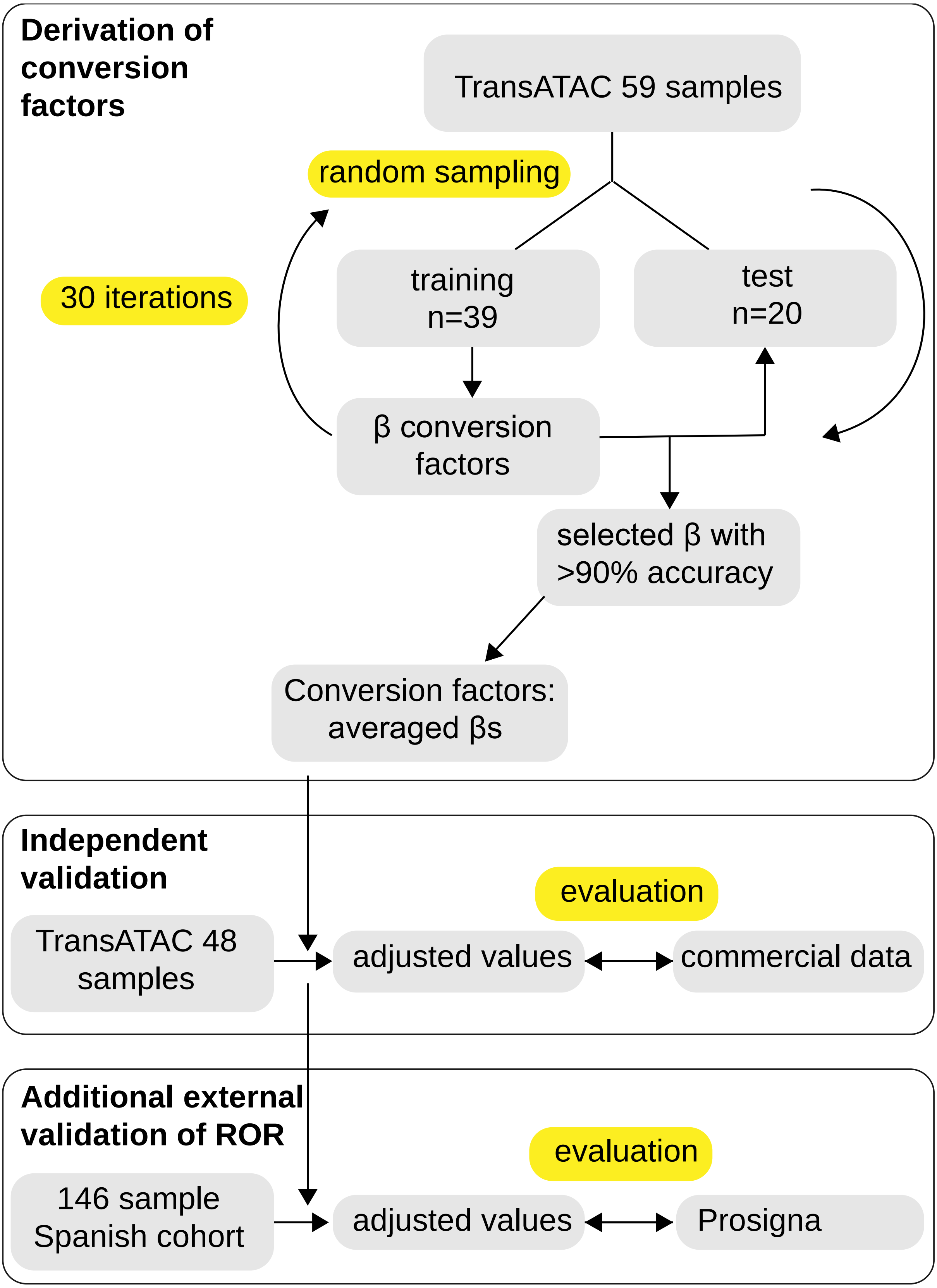

Supplementary Table 1. Agreements between the RT-PCR and the adjusted NanoString gene expression levels for the RS in the validation set (n=48). [a] r_c: concordance correlation coefficient; [b]IQR: interquartile range; [c]LL: lower limit; [d]UL: upper limit; [e]Mean difference of the commercial RT-PCR and adjusted NanoString gene expression levels; [f]SD: standard deviation; [g]LOA: Limits of agreements

		**95% CI[b]**		**Mean**	**SD[f]**	**95% LOA[g]**	
**Gene**	**r_c[a]**	**LL[c]**	**UL[d]**	**difference[e]**	**difference**	**LL**	**UL**
**Proliferation**							
AURKA	0.47	0.22	0.66	-0.01	0.65	-1.29	1.27
BIRC5	0.95	0.92	0.97	0.07	0.32	-0.56	0.69
CCNB1	0.92	0.86	0.95	0.02	0.29	-0.54	0.59
MKI67	0.86	0.78	0.91	0.28	0.37	-0.45	1.00
MYBL2	0.67	0.59	0.75	0.16	0.75	-1.31	1.63
**Estrogen**							
BAG1	0.96	0.94	0.98	-0.13	0.2	-0.51	0.26
BCL2	0.93	0.89	0.96	-0.09	0.42	-0.91	0.74
PGR	0.97	0.94	0.98	0.28	0.46	-0.62	1.18
SCUBE2	1.00	0.99	1.00	-0.08	0.17	-0.41	0.26
**HER2**							
GRB7	0.9	0.84	0.94	0.2	0.25	-0.29	0.69
ERBB2	0.95	0.92	0.97	0.06	0.25	-0.43	0.55
**Invasion**							
CTSL2	0.34	0.12	0.52	0.14	0.71	-1.25	1.52
MMP11	0.97	0.95	0.98	0.08	0.36	-0.64	0.79
**Other**							
GSTM1	0.55	0.38	0.69	-0.15	0.99	-2.09	1.79
CD68	0.91	0.85	0.95	0.01	0.24	-0.47	0.48
BAG1	0.96	0.94	0.98	-0.13	0.2	-0.51	0.26

Supplementary Table 2. Agreements between the RT-PCR and adjusted NanoString gene expression levels for the EP in the validation set (n=24). [a] r_c: concordance correlation coefficient; [b]IQR: interquartile range; [c]LL: lower limit; [d]UL: upper limit; [e]Mean difference of the commercial RT-PCR and adjusted NanoString gene expression levels; [f]SD: standard deviation; [g]LOA: Limits of agreements

Gene	r_c[a]	95% CI[b]		Mean	SD[f]	95% LOA[g]	
		LL[c]	UL[d]	difference[e]	difference.	LL	UL
AZGP1	0.95	0.91	0.97	-0.25	0.31	-0.85	0.35
BIRC5	0.89	0.81	0.93	-0.07	0.57	-1.18	1.04
DHCR7	0.84	0.73	0.9	0.13	0.45	-0.75	1.02
IL6ST	0.94	0.89	0.96	-0.14	0.34	-0.81	0.54
MGP	0.92	0.87	0.95	-0.45	0.49	-1.41	0.5
RBBP8	0.95	0.92	0.97	-0.05	0.34	-0.71	0.61
STC2	0.96	0.92	0.97	-0.03	0.54	-1.1	1.03
UBE2C	0.91	0.88	0.94	0.06	0.4	-0.73	0.84

Supplementary Table 3. Classification of 48 validation set patients by the commercial and RUO RS scores based on two TAILORx-tested cut-points: (a) RS<16 vs RS≥16 and (b) RS<26 vs RS≥26

(a)

Commercial RS (RT-PCR)	RUO RS (NanoString-derived) data		Total
	**<16**	**≥16**	
**<16**	21	3	24
**≥16**	1	23	24
**Total**	22	26	48

(b)

Commercial RS (RT-PCR)	RUO RS (NanoString-derived) data		Total
	**<26**	**≥26**	
**<26**	35	0	35
**≥26**	0	13	13
**Total**	35	13	48

Supplementary Table 4. Breast cancer intrinsic subtype calls by commercial and RUO ROR methods for the TransATAC training and validation sets (n=107).

		**RUO ROR**				
		**Luminal A**	**Luminal B**	**Basal-like**	**HER2-enriched**	**Total**
**Prosigna ROR**	**Luminal A**	49	6	2	1	58
	**Luminal B**	2	29	2	10	43
	**Basal-like**	0	0	0	1	1
	**HER2-enriched**	0	0	0	5	5
	**Total**	51	35	4	17	107

Supplementary Table 5. The custom NanoString gene expression panel used in the TransATAC training (n=59) and validation sets (n=48).

HUGO Gene	Accession	NSID	Target Sequence	RS	ROR	EP	Reference
ACTB	NM_001101.2	NM_001101.2:1685	CCAACTTGAGATGTATGAAGGCTTTTGGTCTCCCTGGGAGTGGGTGGAGGCAGCCAGGGCTTACCTGTACACTGACTTGAGACCAGTTGAATAAAAGTGC	RS	ROR		Reference
ACTR3B	NM_001040135.1	NM_001040135.1:905	CCAGAAGAAGTTTGTaTATAGACGTTGGTTACGAAAGATTCCTGGGACCTGAAATATTCTTTCACCCGGAGTTTGCCAACCCAGACTTTATGGAGTCCATC		ROR		
ANLN	NM_018685.2	NM_018685.2:240	CGTGCCAGGCGAGAGAATCTTCAGAGAAAAATGGCTGAGAGGCCCACAGCAGCTCCAAGGTCTATGACTCATGCTAAGCGAGCTAGACAGCCACTTTCAG		ROR		
AURKA	NM_003600.2	NM_003600.2:405	AGCTCCAGTTGGAGGTCCAAAACGTGTTCTCGTGACTCAGCAATTTCCTTGTCAGAATCCATTACCTGTAAATAGTGGCCAGGCTCAGCGGGTCTTGTGT	RS			
AZGP1	NM_001185.2	NM_001185.2:123	GTTACTCTCTGACCTATATCTACACTGGGCTGTCCAAGCATGTTGAAGACGTCCCCGCGTTTCAGGCCCTTGGCTCACTCAATGACCTCCAGTTCTTTAG			EP	
BAG1	NM_004323.3	NM_004323.3:540	CTTCATGTTACCTCCCAGCAGGGCAGCAGTGAACCAGTTGTCCAAGACCTGGCCCAGGTTGTTGAAGAGGTCATAGGGGTTCCACAGTCTTTTCAGAAAC	RS	ROR		
BCL2	NM_000633.2	NM_000633.2:1525	CCAAGCACCGCTTCGTGTGGCTCCACCTGGATGTTCTGTGCCTGTAAACATAGATTCGCTTTCCATGTTGTTGGCCGGATCACCATCTGAAGAGCAGACG	RS	ROR		
BIRC5	NM_001168.2	NM_001168.2:1215	CCATTCTAAGTCATTGGGGAAACGGGGTGAACTTCAGGTGGATGAGGAGACAGAATAGAGTGATAGGAAGCGTCTGGCAGATACTCCTTTTGCCACTGCT	RS	ROR	EP	
BLVRA	NM_000712.3	NM_000712.3:485	TTCCTGAAAAAAGAAGTGGTGGGGAAAGACCTGCTGAAAGGGTCGCTCCTCTTCACAGCTGGCCCGTTGGAAGAAGAGCGGTTTGGCTTCCCTGCATTCA		ROR		
CALM2	NM_001743.3	NM_001743.3:868	TGGAGTTGTAACTCTGCGTGGACTATGGACAGTCAACAATATGTACTTAAAAGTTGCACTATTGCAAAACGGGTGTATTATCCAGGTACTCGTACACTAT			EP	Reference
CCNB1	NM_031966.2	NM_031966.2:710	GAGACAACTTGAGGAAGAGCAAGCAGTCAGACCAAAATACCTACTGGGTCGGGAAGTCACTGGAAACATGAGAGCCATCCTAATTGACTGGCTAGTACAG	RS	ROR		
CCNE1	NM_001238.1	NM_001238.1:1155	GAGAACTGTGTCAAGTGGATGGTTCCATTTGCCATGGTTATAAGGGAGACGGGGAGCTCAAAACTGAAGCACTTCAGGGGCGTCGCTGATGAAGATGCAC		ROR		
CD68	NM_001251.2	NM_001251.2:1140	ACCGGTCCATCTTGCTGCCTCTCATCATCGGCCTGATCCTTCTTGGCCTCCTCGCCCTGGTGCTTATTGCTTTCTGCATCATCCGGAGACGCCCATCCGC	RS			
CDC20	NM_001255.1	NM_001255.1:915	CCCGAGTGGGCTCCCTAAGCTGGAACAGCTATATCCTGTCCAGTGGTTCACGTTCTGGCCACATCCACCACCATGATGTTCGGGTAGCAGAACACCATGT		ROR		
CDC6	NM_001254.3	NM_001254.3:1655	GGGGAAGTTATATGAAGCCTACAGTAAAGTCTGTCGCAAACAGCAGGTGGCGGCTGTGGACCAGTCAGAGTGTTTGTCACTTTCAGGGCTCTTGGAAGCC		ROR		
CDH3	NM_001793.3	NM_001793.3:2005	CCCTCGACCGTGAGGATGAGCAGTTTGTGAGGAACAACATCTATGAAGTCATGGTCTTGGCCATGGACAATGGAAGCCCTCCCACCACTGGCACGGGAAC		ROR		
CENPF	NM_016343.3	NM_016343.3:9260	AGAAAATCTTGCAGAGTCCTCCAAACCAACAGCTGGTGGCAGCAGATCACAAAAGGTCAAAGTTGCTCAGCGGAGCCCAGTAGATTCAGGCACCATCCTC		ROR		
CEP55	NM_018131.3	NM_018131.3:570	GTACTACCGCATTGCTTGAACAGCTGGAAGAGACAACGAGAGAAGGAGAAAGGAGGGAGCAGGTGTTGAAAGCCTTATCTGAAGAGAAAGACGTATTGAA		ROR		
CTSV	NM_001333.3	NM_001333.3:2820	GTGAAATTTAATCGAAAGGTGATCCATTGTGAATGCAATGGGAGGGAAGGGGCATGTGGGACTGTGTATCCCAAAAACCCTTTGATAGCCTATGTCCACA	RS			
CXXC5	NM_016463.5	NM_016463.5:1630	AGCTGCCCTCTCCGTGCAATGTCACTGCTCGTGTGGTCTCCAGCAAGGGATTCGGGCGAAGACAAACGGATGCACCCGTCTTTAGAACCAAAAATATTCT		ROR		
DHCR7	NM_001360.2	NM_001360.2:780	CCCACCATCATCTTCGACAACTGGATCCCACTGCTGTGGTGCGCCAACATCCTTGGCTATGCCGTCTCCACCTTCGCCATGGTCAAGGGCTACTTCTTCC			EP	

EGFR	NM_005228.3	NM_005228.3:2760	GCAGCCAGGAACGTACTGGTGAAAACACCGCAGCATGTCAAGATCACAGATTTTGGGCTGGCCAAACTGCTGGGTGCGGAAGAGAAAGAATACCATGCAG		ROR		
ERBB2	NM_004448.2	NM_004448.2:2405	TGAAGGTGCTTGGATCTGGCGCTTTTGGCACAGTCTACAAGGGCATCTGGATCCCTGATGGGGAGAATGTGAAAATTCCAGTGGCCATCAAAGTGTTGAG	RS	ROR		
ESR1	NM_000125.2	NM_000125.2:1595	AGGAACCAGGGAAAATGTGTAGAGGGCATGGTGGAGATCTTCGACATGCTGCTGGCTACATCATCTCGGTTCCGCATGATGAATCTGCAGGGAGAGGAGT	RS	ROR		
EXO1	NM_006027.3	NM_006027.3:820	TGGCCCACAAAGTAATTAAAGCTGCCCGGTCTCAGGGGGTAGATTGCCTCGTGGCTCCCTATGAAGCTGATGCGCAGTTGGCCTATCTTAACAAAGCGGG		ROR		
FGFR4	NM_002011.3	NM_002011.3:1002	CCCACATCCAGTGGCTGAAGCACATCGTCATCAACGGCAGCAGCTTCGGAGCCGACGGTTTCCCCTATGTGCAAGTCCTAAAGACTGCAGACATCAATAG		ROR		
FOXA1	NM_004496.2	NM_004496.2:280	TGGATGGTTGTATTGGGCAGGGTGGCTCCAGGATGTTAGGAACTGTGAAGATGGAAGGGCATGAAACCAGCGACTGGAACAGCTACTACGCAGACACGCA		ROR		
FOXC1	NM_001453.1	NM_001453.1:1530	TTCGAGTCACAGAGGATCGGCTTGAACAACTCTCCAGTGAACGGGAATAGTAGCTGTCAAATGGCCTTCCCTTCCAGCCAGTCTCTGTACCGCACGTCCG		ROR		
GAPDH	NM_002046.3	NM_002046.3:972	CACTCCTCCACCTTTGACGCTGGGGCTGGCATTGCCCTCAACGACCACTTTGTCAAGCTCATTTCCTGGTATGACAACGAATTTGGCTACAGCAACAGGG	RS			Reference
GPR160	NM_014373.1	NM_014373.1:760	GGATTTCAGTCCTTGCTTATGTTTTGGGAGACCCAGCCATCTACCAAAGCCTGAAGGCACAGAATGCTTATTCTCGTCACTGTCCTTTCTATGTCAGCAT		ROR		
GRB7	NM_005310.2	NM_005310.2:1010	GCCGATCTGGCCTCTATTACTCCACCAAGGGCACCTCTAAGGATCCGAGGCACCTGCAGTACGTGGCAGATGTGAACGAGTCCAACGTGTACGTGGTGAC	RS	ROR		
GSTM1	NM_000561.2	NM_000561.2:335	GATTCGTGTGGACATTTTGGAGAACCAGACCATGGACAACCATATGCAGCTGGGCATGATCTGCTACAATCCAGAATTTGAGAAACTGAAGCCAAAGTAC	RS			
GUSB	NM_000181.1	NM_000181.1:1350	CGGTCGTGATGTGGTCTGTGGCCAACGAGCCTGCGTCCCACCTAGAATCTGCTGGCTACTACTTGAAGATGGTGATCGCTCACACCAAATCCTTGGACCC	RS	ROR		Reference
IL6ST	NM_002184.2	NM_002184.2:2505	CAAAACACTTCGAGCACTGTCCAGTATTCTACCGTGGTACACAGTGGCTACAGACACCAAGTTCCGTCAGTCCAAGTCTTCTCAAGATCCGAGTCTACCC			EP	
KIF2C	NM_006845.2	NM_006845.2:1020	GTTGTCTACAGGTTCACAGCAAGGCCACTGGTACAGACAATCTTTGAAGGTGGAAAAGCAACTTGTTTTGCATATGGCCAGACAGGAAGTGGCAAGACAC		ROR		
KRT14	NM_000526.3	NM_000526.3:1365	GCAGTCATCCAGAGATGTGACCTCCTCCAGCCGCCAAATCCGCACCAAGGTCATGGATGTGCACGATGGCAAGGTGGTGTCCACCCACGAGCAGGTCCTT		ROR		
KRT17	NM_000422.1	NM_000422.1:1230	CTGACTCAGTACAAGAAAGAACCGGTGACCACCCGTCAGGTGCGTACCATTGTGGAAGAGGTCCAGGATGGCAAGGTCATCTCCTCCCGCGAGCAGGTCC		ROR		
KRT5	NM_000424.2	NM_000424.2:130	CTGGTTCTCTTGCTCCACCAGGAACAAGCCACCATGTCTCGCCAGTCAAGTGTGTCCTTCCGGAGCGGGGGCAGTCGTAGCTTCAGCACCGCCTCTGCCA		ROR		
MAPT	NM_016835.3	NM_016835.3:1425	GCCGGGTCCCTCAACTCAAAGCTCGCATGGTCAGTAAAAGCAAAGACGGGACTGGAAGCGATGACAAAAAAGCCAAGACATCCACACGTTCCTCTGCTAA		ROR		
MDM2	NM_006878.2	NM_006878.2:280	GGTGAGGAGCAGGCAAATGTGCAATACCAACATGTCTGTACCTACTGATGGTGCTGTAACCACCTCACAGATTCCAGCTTCGGAACAAGAGACCCTGGTT		ROR		
MELK	NM_014791.2	NM_014791.2:365	AGAGACAGCCAACAAAATATTCATGGTTCTTGAGTACTGCCCTGGAGGAGAGCTGTTTGACTATATAATTTCCCAGGATCGCCTGTCAGAAGAGGAGACC		ROR		
MGP	NM_000900.2	NM_000900.2:305	TCAATAGGGAAGCCTGTGATGACTACAGACTTTGCGAACGCTACGCCATGGTTTATGGATACAATGCTGCCTATAATCGCTACTTCAGGAAGCGCCGAGG			EP	
MIA	NM_006533.1	NM_006533.1:265	CCGGGGCCAAGTGGTGTATGTCTTCTCCAAGCTGAAGGGCCGTGGGCGGCTCTTCTGGGGAGGCAGCGTTCAGGGAGATTACTATGGAGATCTGGCTGCT		ROR		
MKI67	NM_002417.2	NM_002417.2:2005	GCTTCCAGCAGCAAATCTCAGACAGAGGTTCCTAAGAGAGGAGGAGAAAGAGTGGCAACCTGCCTTCAAAAGAGAGTGTCTATCAGCCGAAGTCAACATG	RS	ROR		
MLPH	NM_024101.4	NM_024101.4:1695	GAGGAAGTCAAACCTCCCGATATTTCTCCCTCGAGTGGCTGGGAAACTTGGCAAGAGACCAGAGGACCCAAATGCAGACCCTTCAAGTGAGGCCAAGGCA		ROR		

MMP11	NM_005940.3	NM_005940.3:702	AGCAGCCAAGGCCCTGATGTCCGCCTTCTACACCTTTCGCTACCCACTGAGTCTCAGCCCAGATGACTGCAGGGGCGTTCAACACCTATATGGCCAGCCC	RS	ROR		
MRPL19	NM_014763.3	NM_014763.3:385	ACAGCTGACCCATATGCCAGTGGAAAAATCAGCCAGTTTCTGGGGATTTGCATTCAGAGATCAGGAAGAGGACTTGGAGCTACTTTCATCCTTAGGAATG		ROR		Reference
MYBL2	NM_002466.2	NM_002466.2:675	GCAACCGCTGGGCCGAGATCGCCAAGATGTTGCCAGGGAGGACAGACAATGCTGTGAAGAATCACTGGAACTCTACCATCAAAAGGAAGGTGGACACAGG	RS	ROR		
MYC	NM_002467.3	NM_002467.3:1615	CACCGAGGAGAATGTCAAGAGGCGAACACACAACGTCTTGGAGCGCCAGAGGAGGAACGAGCTAAAACGGAGCTTTTTTGCCCTGCGTGACCAGATCCCG		ROR		
NAT1	NM_000662.4	NM_000662.4:0	AGCACTTCCTCATAGACCTTGGATGTGGGAGGATTGCATTCAGTCTAGTTCCTGGTTGCCGGCTGAAATAACCTGAATTCAAGCCAGGAAGAAGCAGCAA		ROR		
NDC80	NM_006101.1	NM_006101.1:90	AAAAGGTCATAAGCATGAAGCGCAGTTCAGTTTCCAGCGGTGGTGCTGGCCGCCTCTCCATGCAGGAGTTAAGATCCCAGGATGTAAATAAACAAGGCCT		ROR		
NUF2	NM_145697.1	NM_145697.1:215	GCCTGGCGGTGTTTTCGTCGTGCTCAGCGGTGGGAGGAGGCGGAAGAAACCAGAGCCTGGGAGATTAACAGGAAACTTCCAAGATGGAAACTTTGTCTTT		ROR		
OAZ1	NM_004152.2	NM_004152.2:313	GGTGGGCGAGGGAATAGTCAGAGGGATCACAATCTTTCAGCTAACTTATTCTACTCCGATGATCGGCTGAATGTAACAGAGGAACTAACGTCCAACGACA			EP	Reference
ORC6	NM_014321.2	NM_014321.2:580	GACTGTGTAAACAACTAGAGAAGATTGGACAGCAGGTCGACAGAGAACCTGGAGATGTAGCTACTCCACCACGGAAGAGAAAGAAGATAGTGGTTGAAGC		ROR		
PGR	NM_000926.2	NM_000926.2:3165	GGGATGAAGCATCAGGCTGTCATTATGGTGTCCTTACCTGTGGGAGCTGTAAGGTCTTCTTTAAGAGGGCAATGGAAGGGCAGCACAACTACTTATGTGC	RS	ROR		
PHGDH	NM_006623.2	NM_006623.2:505	GCGACGGCTTCGATGAAGGACGGCAAATGGGAGCGGAAGAAGTTCATGGGAACAGAGCTGAATGGAAAGACCCTGGGAATTCTTGGCCTGGGCAGGATTG		ROR		
PSMC4	NM_006503.2	NM_006503.2:300	CATCGGACAATTTCTGGAGGCTGTGGATCAGAATACAGCCATCGTGGGCTCTACCACAGGCTCCAACTATTATGTGCGCATCCTGAGCACCATCGATCGG		ROR		Reference
PTTG1	NM_004219.2	NM_004219.2:202	CACCAGCCTTACCTAAAGCTACTAGAAAGGCTTTGGGAACTGTCAACAGAGCTACAGAAAAGTCTGTAAAGACCAAGGGACCCCTCAAACAAAAACAGCC		ROR		
PUM1	NM_001020658.1	NM_001020658.1:640	CTGGGGAACATCAGATCATTCAGTTTCCCAGCCAATCATGGTGCAGAGAAGACCTGGTCAGAGTTTCCATGTGAACAGTGAGGTCAATTCTGTACTGTCC		ROR		Reference
RBBP8	NM_002894.2	NM_002894.2:760	AATGATCAACAGCATCAAGCAGCTGAGCTTGAATGTGAGGAAGACGTTATTCCAGATTCACCGATAACAGCCTTCTCATTTTCTGGCGTTAACCGGCTAC			EP	
RPL37A	NM_000998.4	NM_000998.4:298	CTTCCGCTGTCACGGTAAAGTCCGCCATCAGAAGACTGAAGGAGTTGAAAGACCAGTAGACGCTCCTCTACTCTTTGAGACATCACTGGCCTATAATAAA			EP	Reference
RPLP0	NM_001002.3	NM_001002.3:250	CGAAATGTTTCATTGTGGGAGCAGACAATGTGGGCTCCAAGCAGATGCAGCAGATCCGCATGTCCCTTCGCGGGAAGGCTGTGGTGCTGATGGGCAAGAA	RS	ROR		Reference
RRM2	NM_001034.1	NM_001034.1:490	TTCCTTTTGGACCGCCGAGGAGGTTGACCTCTCCAAGGACATTCAGCACTGGGAATCCCTGAAACCCGAGGAGAGATATTTTATATCCCATGTTCTGGCT		ROR		
SCUBE2	NM_020974.1	NM_020974.1:1835	CGTAAAGCCATCCGCACGCTCAGAAAGGCCGTCCACAGGGAGCAGTTTCACCTCCAGCTCTCAGGCATGAACCTCGACGTGGCTAAAAAGCCTCCCAGAA	RS			
SF3A1	NM_005877.4	NM_005877.4:1485	GATGATGAGGTGTACGCACCAGGTCTGGATATTGAGAGCAGCTTGAAGCAGTTGGCTGAGCGGCGTACTGACATCTTCGGTGTAGAGGAAACAGCCATTG		ROR		Reference
SFRP1	NM_003012.3	NM_003012.3:1320	GTGGGTCACACACACGCACTGCGCCTGTCAGTAGTGGACATTGTAATCCAGTCGGCTTGTTCTTGCAGCATTCCCGCTCCCTTCCCTCCATAGCCACGCT		ROR		
SLC39A6	NM_012319.2	NM_012319.2:1580	GATCGAACTGAAGGCTATTTACGAGCAGACTCACAAGAGCCCTCCCACTTTGATTCTCAGCAGCCTGCAGTCTTGGAAGAAGAAGAGGTCATGATAGCTC		ROR		
STC2	NM_003714.2	NM_003714.2:2825	ATTTCTATGTGTAATTTCTGAGCCATTGTACTGTCTGGGCTGGGGGGGACACTGTCCAAGGGAGTGGCCCCTATGAGTTTATATTTTAACCACTGCTTCA			EP	
TFRC	NM_003234.1	NM_003234.1:1220	CAGTTTCCACCATCTCGGTCATCAGGATTGCCTAATATACCTGTCCAGACAATCTCCAGAGCTGCTGCAGAAAAGCTGTTTGGGAATATGGAAGGAGACT	RS	ROR		Reference

TMEM45B	NM_138788.3	NM_138788.3:730	CTGGCTGCCCTCAGCATTGTGGCCGTCAACTATTCTCTTGTTTACTGCCTTTTGACTCGGATGAAGAGACACGGAAGGGGAGAAATCATTGGAATTCAGA		ROR		
TYMS	NM_001071.1	NM_001071.1:395	TGCTAAAGAGCTGTCTTCCAAGGGAGTGAAAATCTGGGATGCCAATGGATCCCGAGACTTTTTGGACAGCCTGGGATTCTCCACCAGAGAAGAAGGGGAC		ROR		
UBE2C	NM_007019.2	NM_007019.2:445	GTCTGCCCTGTATGATGTCAGGACCATTCTGCTCTCCATCCAGAGCCTTCTAGGAGAACCCAACATTGATAGTCCCTTGAACACACATGCTGCCGAGCTC		ROR	EP	
UBE2T	NM_014176.1	NM_014176.1:50	GTGTCAGCTCAGTGCATCCCAGGCAGCTCTTAGTGTGGAGCAGTGAACTGTGTGTGGTTCCTTCTACTTGGGGATCATGCAGAGAGCTTCACGTCTGAAG		ROR		

Supplementary Table 6. Normalsation factors used to adjust expression values in order to calculate RUO ROR in TransATAC samples. These factors were calculated by scaling the 59 TransATAC training set samples to the 229 sample ER+/HER2- tumours (ERPosHER2Neg set) previously subjected to the Prosigna® assay.

Gene	Cohort median adjustment factors
ACTR3B	0.063
ANLN	-1.043
BAG1	0.325
BCL2	0.362
BLVRA	0.421
CCNE1	-0.78
CDC20	-0.604
CDC6	0.093
CDCA1	0.242
CDH3	-0.019
CENPF	-0.641
CEP55	-0.715
CXXC5	0.732
EGFR	0.471
ERBB2	1.034
ESR1	1.555
EXO1	-0.374
FGFR4	-0.98
FOXA1	-0.108
FOXC1	-0.027
GPR160	0.633
KIF2C	0.779

KNTC2	-0.2
KRT14	0.981
KRT17	1.715
KRT5	0.672
MAPT	0.831
MDM2	-0.167
MELK	-0.024
MIA	1.076
MKI67	0.013
MLPH	0.761
MMP11	0.404
MYC	-0.362
NAT1	1.066
ORC6L	-0.127
PGR	0.86
PHGDH	-0.099
PTTG1	-1.386
RRM2	-1.105
SFRP1	0.078
SLC39A6	0.821
TMEM45B	-0.082
TYMS	-0.619
UBE2C	-0.563
UBE2T	-0.023

Supplementary Table 7. Calibration factors to adjust for microarray derived centroids

	PAM50RUO calibration factor
ACTR3B	-3.0222214
ANLN	-3.9565307

BAG1	-1.485749
BCL2	-3.0901392
BIRC5	-2.5264471
BLVRA	-1.7121774
CCNB1	-2.6797027
CCNE1	-5.0101326
CDC20	-4.6649842
CDC6	-3.8592176
CDCA1	-6.285376
CDH3	-3.9795265
CENPF	-2.9930417
CEP55	-4.1826523
CXXC5	-1.4574313
EGFR	-5.3645631
ERBB2	-1.910135
ESR1	-1.3917915
EXO1	-4.8670732
FGFR4	-5.1261057
FOXA1	-1.0402642
FOXC1	-5.1657298
GPR160	-1.8332197
GRB7	-4.5871578
KIF2C	-6.0513213
KNTC2	-4.7378452
KRT14	-3.216056
KRT17	-4.1054272
KRT5	-4.8900259
MAPT	-3.994877
MDM2	-0.9628511
MELK	-4.6168289
MIA	-7.7403848
MKI67	-4.0156607
MLPH	-1.1954999

MMP11	-1.3008179
MYBL2	-4.7857388
MYC	-0.8680514
NAT1	-3.3472933
ORC6L	-5.2980294
PGR	-6.014928
PHGDH	-3.1518718
PTTG1	-3.2230568
RRM2	-2.4655345
SFRP1	-3.3553648
SLC39A6	-0.6547639
TMEM45B	-6.9167267
TYMS	-3.0764834
UBE2C	-2.7563288
UBE2T	-4.5865595

Supplementary Table 8. Centroids representing the Basal-like, HER2-enriched, Luminal A, Luminal B and Normal-like.subtypes**.**

	**Basal**	**Her2**	**LumA**	**LumB**	**Normal**
ACTR3B	0.71833189	-0.4816657	0.00998107	-0.1905513	0.46572287
ANLN	0.5373723	0.26693161	-0.5792457	0.09880418	-0.8369396
BAG1	-0.5745069	-0.4760729	0.75822116	-0.4054586	0.31655297
BCL2	-0.1187604	-0.157914	0.28748744	-0.4413395	0.53397887
BLVRA	-0.6426775	0.33533604	0.04204202	0.69120496	-0.1634128
CCNE1	0.56027103	0.06687223	-0.4302912	-0.0166614	-0.2554761
CDC20	0.39969524	0.00835552	-0.469044	-0.0704125	-0.0455048
CDC6	0.15941828	0.58900682	-0.6128243	0.51089597	-0.5957522
CDCA1	0.47240017	-0.0238192	-0.7125208	0.58962688	-0.3705334
CDH3	0.50836201	0.21088969	-0.5136493	-1.4191344	0.75792062

CENPF	0.48297629	-0.0292662	-0.5437402	0.27822856	-0.0705831
CEP55	0.56774889	0.27638102	-0.7467217	0.46001576	-1.1623742
CXXC5	-0.9203858	-0.2415506	0.46741157	0.32133502	0.05090144
EGFR	-0.0304168	-0.0963826	0.00916296	-0.4124013	0.34163708
ERBB2	-0.808354	1.75984423	0.60819126	0.15965187	-0.8702385
ESR1	-2.7465131	-1.5131113	2.16141188	1.60589991	-0.4182823
EXO1	0.42809036	0.04929719	-0.5674745	0.14124128	-0.4507805
FGFR4	-0.271238	0.82177815	0.17081193	-0.247036	0.85747278
FOXA1	-2.6269467	0.02282715	1.01745742	0.3607578	-0.7828121
FOXC1	1.49045147	-0.9471742	-0.174958	-1.564855	1.11154786
GPR160	-1.0549747	0.58319483	0.68548997	0.7144076	-0.4235685
KIF2C	0.20357258	-0.165102	-0.5053947	-0.1828907	-0.3900145
KNTC2	0.60035617	0.04254679	-0.588221	0.38670684	-1.0696289
KRT14	0.09682672	-0.4436461	0.36837594	-0.639447	1.73568631
KRT17	0.48256553	-0.3378371	0.01420986	-1.4637429	1.75959844
KRT5	0.50664042	-0.4282618	0.21532007	-0.9116073	1.7851169
MAPT	-0.4258293	-0.3575065	0.70062272	-0.1903406	0.1178285
MDM2	-0.2513662	-0.1067287	0.14195743	-0.133779	0.27421401
MELK	0.52303387	0.19801312	-0.5820881	0.44793463	-0.7437647
MIA	1.57827637	-0.9048986	-0.1652586	-1.4229263	2.03885956
MKI67	0.47653745	0.06566236	-0.5018716	-0.1452179	-0.1660041
MLPH	-0.3399725	-0.1952287	0.33930442	-0.4561499	0.75075837
MMP11	-0.5560377	0.50675876	-0.0062551	0.33419931	-2.3269851
MYC	0.17876381	-1.0468328	-0.0908308	0.0152644	1.0291762
NAT1	-0.9368489	-0.0899885	2.92278679	0.47078804	-0.3632738
ORC6L	0.2163048	0.20440245	-0.3522207	0.11062765	-0.2558795
PGR	-0.4291334	-0.2794099	0.445785	-0.4488398	0.12601148
PHGDH	0.63451887	-0.1866259	-0.3986822	-1.0301393	0.66043775
PTTG1	0.26413189	0.0558099	-0.6344683	0.24972528	-0.5497813
RRM2	0.15620468	0.68272489	-0.9507602	0.35066384	-1.1210549
SFRP1	0.98798846	-1.0482027	0.13156636	-1.7204583	2.43628867
SLC39A6	-1.0511251	-0.6957365	2.06145908	1.65330302	0.11688969
TMEM45B	-1.1094582	1.33063617	0.44624205	0.37568823	0.03620891

TYMS	0.4498009	0.0529449	-0.6446021	0.49260652	-0.7269895
UBE2C	0.21853415	0.0610806	-0.5198184	0.29279931	-0.4088947
UBE2T	0.3899089	0.28453681	-0.5392594	0.73895213	-0.952381
