# Supplementary material for: Development and validation for research assessment of Oncotype DX® Breast Recurrence Score, EndoPredict® and Prosigna®
Source: NPJ Breast Cancer. 2021 Feb 12;7:15. doi: 10.1038/s41523-021-00216-w (PMC7881187; doi:10.1038/s41523-021-00216-w)
Supplement: Supplementary file 3 — Reporting summary. [file 41523_2021_216_MOESM3_ESM.pdf]

nature research

Corresponding author(s): Maggie Cheang

Last updated by author(s): Dec 11, 2020

Reporting Summary

Nature Research wishes to improve the reproducibility of the work that we publish. This form provides structure for consistency and transparency in reporting. For further information on Nature Research policies, see our Editorial Policies and the Editorial Policy Checklist.

Statistics

For all statistical analyses, confirm that the following items are present in the figure legend, table legend, main text, or Methods section.

n/a	Confirmed	
☐	☒	The exact sample size (*n*) for each experimental group/condition, given as a discrete number and unit of measurement
☐	☒	A statement on whether measurements were taken from distinct samples or whether the same sample was measured repeatedly
☐	☒	The statistical test(s) used AND whether they are one- or two-sided<br>*Only common tests should be described solely by name; describe more complex techniques in the Methods section.*
☐	☒	A description of all covariates tested
☐	☒	A description of any assumptions or corrections, such as tests of normality and adjustment for multiple comparisons
☐	☒	A full description of the statistical parameters including central tendency (e.g. means) or other basic estimates (e.g. regression coefficient) AND variation (e.g. standard deviation) or associated estimates of uncertainty (e.g. confidence intervals)
☐	☒	For null hypothesis testing, the test statistic (e.g. *F*, *t*, *r*) with confidence intervals, effect sizes, degrees of freedom and *P* value noted<br>*Give P values as exact values whenever suitable.*
☒	☐	For Bayesian analysis, information on the choice of priors and Markov chain Monte Carlo settings
☒	☐	For hierarchical and complex designs, identification of the appropriate level for tests and full reporting of outcomes
☐	☒	Estimates of effect sizes (e.g. Cohen's *d*, Pearson's *r*), indicating how they were calculated

Our web collection on statistics for biologists contains articles on many of the points above.

Software and code

Policy information about availability of computer code

Data collection	STATA 15.0, EXCEL 2016
Data analysis	STATA 15.0 and R 3.6.1 were used for statistical calculations.

For manuscripts utilizing custom algorithms or software that are central to the research but not yet described in published literature, software must be made available to editors and reviewers. We strongly encourage code deposition in a community repository (e.g. GitHub). See the Nature Research guidelines for submitting code & software for further information.

Data

Policy information about availability of data

All manuscripts must include a data availability statement. This statement should provide the following information, where applicable:
- Accession codes, unique identifiers, or web links for publicly available datasets
- A list of figures that have associated raw data
- A description of any restrictions on data availability

Data subject to third party restrictions.

Field-specific reporting

Please select the one below that is the best fit for your research. If you are not sure, read the appropriate sections before making your selection.

☒ Life sciences ☐ Behavioural & social sciences ☐ Ecological, evolutionary & environmental sciences

For a reference copy of the document with all sections, see nature.com/documents/nr-reporting-summary-flat.pdf

Life sciences study design

All studies must disclose on these points even when the disclosure is negative.

Sample size

Our analytical approach is to compute conversion factors by modelling the gene expression data values measured by nanostring with those assessed by the commercial assays (Supplemental Figure 10). Stratified by the risk groups, tumour size and nodal status, we randomly split the 107 cases into training set (n=59) for development and validation set (n=48) to determine the correlation coefficient between the Research Use Only and their commercial scores. In order to detect a positive relationship (i.e. correlation >0.4) relationship with 85% power and 0.05 significance level, we would need at least 42 cases. Furthermore, as a secondary analysis, 48 cases would also have approximately 86% power to detect 20% variability of the model at a 0.05 significance level.

Data exclusions

No data was excluded from the analyses

Replication

This study is a computational methodological paper,as described above, on computing computing coversion factors by modelling the gene expression data values measured by nanostring with those assessed by the commercial assays. Therefore, not a biological experimental study re replicating the findings. On the other hand, we apply a cross-validation analytical approach. Stratified by the risk groups, tumour size and nodal status, we randomly split the 107 cases into training set (n=59) for development and validation set (n=48) to determine the correlation coefficient between the Research Use Only and their commercial scores. We also identify an independent cohort of patients (n = 143) to replicate the results of research use only ROR scores.

Randomization

This is not a clinical trial study or clinical studies to evaluate the treatment effect, which usually this is about. Meanwhile the randomisation procedure, described above, study samples were randomised into training and validation test stratified by risk groups, tuomur size and nodal status.

Blinding

Data analyst were blinded to the patient ID and survival clinical outcome. Data analyst and bioinformaticians had access to the commercial score results of the training set data. For the external validation and the validation dataset the analyst were completely blinded to the commercial score results when applying the algorithms, and only had access the "gold standard: commercial scores at the final step to determine the agreements.

Reporting for specific materials, systems and methods

We require information from authors about some types of materials, experimental systems and methods used in many studies. Here, indicate whether each material, system or method listed is relevant to your study. If you are not sure if a list item applies to your research, read the appropriate section before selecting a response.

Materials & experimental systems

n/a	Involved in the study
☒	☐ Antibodies
☒	☐ Eukaryotic cell lines
☒	☐ Palaeontology and archaeology
☒	☐ Animals and other organisms
☒	☐ Human research participants
☒	☐ Clinical data
☒	☐ Dual use research of concern

Methods

n/a	Involved in the study
☒	☐ ChIP-seq
☒	☐ Flow cytometry
☒	☐ MRI-based neuroimaging
